# Supplementary material for: Cold-responsive transcription factors in Arabidopsis and rice: A regulatory network analysis using array data and gene co-expression network
Source: PLoS One. 2023 Jun 8;18(6):e0286324. doi: 10.1371/journal.pone.0286324 (PMC10249815; doi:10.1371/journal.pone.0286324)
Supplement: S1 Table — (DOCX) [file pone.0286324.s001.docx]

| **Supplementary Table S1**: Accession number of *A. thaliana* and *O. sativa* Two-week old seedlings microarray from GEO | | | | |
| --- | --- | --- | --- | --- |
| Reference | Cold treatment | Accession Number | No. | Plant name |
| [117] | 4° C | GSE33978 | 1 | Arabidopsis |
| [118] | 4°C | GSE63184 | 2 |  |
| [119] | 4°C | GSE5536 | 3 |  |
| [120] | 0 C | GSE3326 | 4 |  |
| [121] | 4°C | GSE86605 | 5 |  |
| [122] | 0 C | GSE63131 | 6 |  |
| [123] | 4°C | GSE41935 | 7 |  |
| [124] | 4°C | GSE38030 | 8 |  |
| [125] | 4°C | GSE38023 | 1 | Rice |
| [126] | 4°C | GSE71680 | 2 |  |
| [127] | 4°C | GSE83912 | 3 |  |
| [128] | 4°C | GSE37940 | 4 |  |
| [129] | 5°C | GSE32065 | 5 |  |
| [130] | 4°C | GSE19983 | 6 |  |
| [131] | 4°C | GSE32704 | 7 |  |
| [132] | 4°C | GSE6901 | 8 |  |

References

117.Hatano-Iwasaki A, Ogawa K. Overexpression of GSH1 gene mimics transcriptional response to low temperature during seed vernalization treatment of Arabidopsis. Plant Cell Physiol. 2012;53(7):1195-203. doi: 10.1093/pcp/pcs075.

118.Chan Z, Wang Y, Cao M, Gong Y, Mu Z, Wang H, Hu Y, Deng X, He XJ, Zhu JK. RDM4 modulates cold stress resistance in Arabidopsis partially through the CBF-mediated pathway. New Phytol. 2016;209(4):1527-39. doi: 10.1111/nph.13727. Epub 2015 Nov 2. PMID: 26522658; PMCID: PMC5515388.

119.[Vogel J](https://www.ncbi.nlm.nih.gov/pubmed/?term=Vogel%20J%5bAuthor%5d), [Thomashow M](https://www.ncbi.nlm.nih.gov/pubmed/?term=Thomashow%20M%5bAuthor%5d), [Townsend H](https://www.ncbi.nlm.nih.gov/pubmed/?term=Townsend%20H%5bAuthor%5d), [Emmerson Z](https://www.ncbi.nlm.nih.gov/pubmed/?term=Emmerson%20Z%5bAuthor%5d), [Schildknecht B](https://www.ncbi.nlm.nih.gov/pubmed/?term=Schildknecht%20B%5bAuthor%5d). [GSE5536](https://www.ncbi.nlm.nih.gov/geo/query/acc.cgi?acc=GSE5536)**. 2006. https://www.ncbi.nlm.nih.gov/geo/query/acc.cgi.**

120. Lee BH, Henderson DA, Zhu JK. The Arabidopsis cold-responsive transcriptome and its regulation by ICE1. Plant Cell. 2005;17(11):3155-75. doi: 10.1105/tpc.105.035568.

121.Eremina M, Unterholzner SJ, Rathnayake AI, Castellanos M, Khan M, Kugler KG, May ST, Mayer KF, Rozhon W, Poppenberger B. Brassinosteroids participate in the control of basal and acquired freezing tolerance of plants. Proc Natl Acad Sci U S A. 2016;113(40):E5982-E5991. doi: 10.1073/pnas.1611477113.

122.Song Y, Liu L, Li G, An L, Tian L. Trichostatin A and 5-Aza-2'-Deoxycytidine influence the expression of cold-induced genes in Arabidopsis. Plant Signal Behav. 2017;12(11):e1389828. doi: 10.1080/15592324.2017.1389828.

123.Rasmussen S, Barah P, Suarez-Rodriguez MC, Bressendorff S, Friis P, Costantino P, Bones AM, Nielsen HB, Mundy J. Transcriptome responses to combinations of stresses in Arabidopsis. Plant Physiol. 2013;161(4):1783-94. doi: 10.1104/pp.112.210773.

124. Juntawong P, Sorenson R, Bailey-Serres J. Cold shock protein 1 chaperones mRNAs during translation in Arabidopsis thaliana. Plant J. 2013;74(6):1016-28. doi: 10.1111/tpj.12187..

125. Zhang T, Zhao X, Wang W, Pan Y, Huang L, Liu X, Zong Y, Zhu L, Yang D, Fu B. Comparative transcriptome profiling of chilling stress responsiveness in two contrasting rice genotypes. PLoS One. 2012;7(8):e43274. doi: 10.1371/journal.pone.0043274.

126.Lv Y, Guo Z, Li X, Ye H, Li X, Xiong L. New insights into the genetic basis of natural chilling and cold shock tolerance in rice by genome-wide association analysis. Plant Cell Environ. 2016;39(3):556-70. doi: 10.1111/pce.12635.

127.Lv Y, Yang M, Hu D, Yang Z, Ma S, Li X, Xiong L. The OsMYB30 Transcription Factor Suppresses Cold Tolerance by Interacting with a JAZ Protein and Suppressing β-Amylase Expression. Plant Physiol. 2017;173(2):1475-1491. doi: 10.1104/pp.16.01725.

128. Zhang F, Huang L, Wang W, Zhao X, Zhu L, Fu B, Li Z. Genome-wide gene expression profiling of introgressed indica rice alleles associated with seedling cold tolerance improvement in a japonica rice background. BMC Genomics. 2012;13:461. doi: 10.1186/1471-2164-13-461.

129.Park SH, Chung PJ, Juntawong P, Bailey-Serres J, Kim YS, Jung H, Bang SW, Kim YK, Do Choi Y, Kim JK. Posttranscriptional control of photosynthetic mRNA decay under stress conditions requires 3' and 5' untranslated regions and correlates with differential polysome association in rice. Plant Physiol. 2012;159(3):1111-24. doi: 10.1104/pp.112.194928.

130. [Mittal D](https://www.ncbi.nlm.nih.gov/pubmed/?term=Mittal%20D%5bAuthor%5d), [Grover A](https://www.ncbi.nlm.nih.gov/pubmed/?term=Grover%20A%5bAuthor%5d). 2012. GSE19983. https://www.ncbi.nlm.nih.gov/geo/query/acc.cgi.

131.Mittal D, Madhyastha DA, Grover A. Gene expression analysis in response to low and high temperature and oxidative stresses in rice: combination of stresses evokes different transcriptional changes as against stresses applied individually. Plant Sci. 2012;197:102-13. doi: 10.1016/j.plantsci.2012.09.008.

132.Arora R, Agarwal P, Ray S, Singh AK, Singh VP, Tyagi AK, Kapoor S. MADS-box gene family in rice: genome-wide identification, organization and expression profiling during reproductive development and stress. BMC Genomics. 2007;18(8)242. doi: 10.1186/1471-2164-8-242.
