# Supplementary material for: Cold-responsive transcription factors in Arabidopsis and rice: A regulatory network analysis using array data and gene co-expression network
Source: PLoS One. 2023 Jun 8;18(6):e0286324. doi: 10.1371/journal.pone.0286324 (PMC10249815; doi:10.1371/journal.pone.0286324)
Supplement: S2 Table — (DOCX) [file pone.0286324.s002.docx]

| **Supplementary Table S2**: Uncommon up- and down-regulated TFs in Rice and Arabidopsis | | | | | | | | | | | | |
| --- | --- | --- | --- | --- | --- | --- | --- | --- | --- | --- | --- | --- |
| TF Family | Rice up | | | Rice down | | | | Arabidopsis up | | | | Arabidopsis down |
| AP2-like ethylene-responsive | WRI4(At1g79700() | | | (WRI3) At1g16060 | | | | SMZ(At3g54990) | | | | PLT1(AT3G20840) |
|  | PLT1(AT3G20840) | | |  | | | | AIL6 (At5g10510) | | | | BBM(AT5G17430) |
|  | BBM(AT5G17430) | | |  | | | | TOE2(At5g60120) | | | |  |
| Ethylene-responsive (ERF) | DREB 2D | | | ERF3 | | | | DREB2A | | | | SHINE 3 |
|  | DREB 1E | | | ERF038 | | | | DREB A-4 | | | | ABI4 |
|  | DREB 1G | | | ERF054 | | | | DREB2B | | | | RAP2-10 |
|  | ERF8 | | | ERF086 | | | | DREB1D/CBF4 | | | | ERF011 |
|  | ERF20 | | | ERF073 | | | | DREB1C/CBF2 | | | | ERF024 |
|  | ERF27 | | | ERF105 | | | | DDF2 | | | | ERF025 |
|  | ERF109 | | | ERF110 | | | | RAP2-1 | | | | ERF35 |
|  | ERF117 | | | WIN1 | | | | RAP2-11 | | | | ERF36 |
|  | ERF118 | | | LEP | | | | ERF3 | | | | ERF060 |
|  | RAP2-13 | | |  | | | | ERF6 | | | | ERF095 |
|  | RAP2-3 | | |  | | | | ERF9 | | | | ERF098 |
|  | RAP2-9 | | |  | | | | ERF10 | | | | ERF104 |
|  | ABR1 | | |  | | | | ERF11 | | | | ERF112 |
|  | ERF-1B | | |  | | | | ERF12 | | | |  |
|  | CRF5 | | |  | | | | ERF19 | | | |  |
|  |  | | |  | | | | ERF22 | | | |  |
|  |  | | |  | | | | ERF34 | | | |  |
|  |  | | |  | | | | ERF55 | | | |  |
|  |  | | |  | | | | ERF56 | | | |  |
|  |  | | |  | | | | ERF57 | | | |  |
|  |  | | |  | | | | ERF 61 | | | |  |
|  |  | | |  | | | | ERF106 | | | |  |
|  |  | | |  | | | | ERF107 | | | |  |
| MYB | APL | | | MYB30 | | | | MYB9 | | | | MYB125 (DUO1) |
|  | Myb4-like | | | MYB 18(LAF1) | | | | MYB10 | | | | MYB-related ASG4 (AT1G01520) |
|  | MYB39 | | |  | | | | MYB18 | | | | MYB-related TRY |
|  | MYB122 | | |  | | | | MYB19 | | | | MYB72 |
|  | GAMYB | | |  | | | | MYB25 | | | | MYB8 (HOS10) |
|  |  | | |  | | | | MYB37 | | | | MYB102 |
|  |  | | |  | | | | MYB52 | | | |  |
|  |  | | |  | | | | MYB53 | | | |  |
|  |  | | |  | | | | MYB61 | | | |  |
|  |  | | |  | | | | MYB62 | | | |  |
|  |  | | |  | | | | MYB65 | | | |  |
|  |  | | |  | | | | MYB72 | | | |  |
|  |  | | |  | | | | MYB76 | | | |  |
|  |  | | |  | | | | MYB84 | | | |  |
|  |  | | |  | | | | MYB87 | | | |  |
|  |  | | |  | | | | MYB92 | | | |  |
|  |  | | |  | | | | MYB93 | | | |  |
|  |  | | |  | | | | MYB97 | | | |  |
|  |  | | |  | | | | MYB110 | | | |  |
|  |  | | |  | | | | MYB114 | | | |  |
|  |  | | |  | | | | MYB117/ LOF1 | | | |  |
|  |  | | |  | | | | MYB118 | | | |  |
|  |  | | |  | | | | MYB119, MYB123 (TT2) | | | |  |
|  |  | | |  | | | | RVE1 | | | |  |
|  |  | | |  | | | | Circadian 1/ RVE2 | | | |  |
|  |  | | |  | | | | TRFL6 | | | |  |
|  |  | | |  | | | | RSM1/ MEE3 | | | |  |
|  |  | | |  | | | | MYB- AS1 | | | |  |
|  |  | | |  | | | | RAX2 | | | |  |
|  |  | | |  | | | | AT1G19000 | | | |  |
|  |  | | |  | | | | AT1G74840 | | | |  |
| **Supplementary Table S2**: Uncommon up- and down-regulated TFs in Rice and Arabidopsis | | | | | | | | | | | | |
| TF Family | | | Rice up | | | Rice down | | | Arabidopsis up | Arabidopsis down | | |
| bHLH | | | bHLH13 | | | bHLH18 | | | bHLH 3 | bHLH15, PIF1 (PIL5) | | |
|  |  |  | bHLH96 | | | bHLH30 | | | bHLH 4(AT4G17880) | bHLH71 | | |
|  |  |  | bHLH 32 (AIG1) | | | bHLH47 | | | bHLH7 | bHLH88 (HEC1) | | |
|  |  |  | bHLH 37 (HEC2) | | | bHLH48 | | | bHLH 12 (ATMYC1) | bHLH101 | | |
|  |  |  |  | | | bHLH49 | | | bHLH18 | bHLH122 | | |
|  |  |  |  | | | bHLH62 | | | bHLH30 | bHLH126 | | |
|  |  |  |  | | | bHLH63 | | | bHLH32 | POPEYE | | |
|  |  |  |  | | | bHLH68 | | | bHLH34 | bHLH- BPE(AT1G59640) | | |
|  |  |  |  | | | bHLH77 | | | bHLH 42 (TT8) |  | | |
|  |  |  |  | | | bHLH83 | | | bHLH 45 (MUTE) |  | | |
|  |  |  |  | | | bHLH94 | | | bHLH48 |  | | |
|  |  |  |  | | | bHLH96 | | | bHLH57 |  | | |
|  |  |  |  | | | bHLH110 | | | bHLH60 |  | | |
|  |  |  |  | | | bHLH121 | | | bHLH62 |  | | |
|  |  |  |  | | | bHLH130 | | | bHLH64 |  | | |
|  |  |  |  | | | bHLH140 | | | bHLH67 |  | | |
|  |  |  |  | | | bHLH144 | | | bHLH68, |  | | |
|  |  |  |  | | | bHLH150 | | | bHLH71, |  | | |
|  |  |  |  | | | ILI2 | | | bHLH74, |  | | |
|  |  |  |  | | | ILI4 | | | bHLH78 |  | | |
|  |  |  |  | | | ILI6 | | | bHLH89 |  | | |
|  |  |  |  | | | bHLH 20 (NAI1) | | | bHLH91 |  | | |
|  |  |  |  | | |  | | | bHLH 97 (FAMA) |  | | |
|  |  |  |  | | |  | | | bHLH99 |  | | |
|  |  |  |  | | |  | | | bHLH104 |  | | |
|  |  |  |  | | |  | | | bHLH109 |  | | |
|  |  |  |  | | |  | | | bHLH114 |  | | |
|  |  |  |  | | |  | | | bHLH115 |  | | |
|  |  |  |  | | |  | | | bHLH121 |  | | |
|  |  |  |  | | |  | | | bHLH130 |  | | |
|  |  |  |  | | |  | | | bHLH133 |  | | |
|  |  |  |  | | |  | | | bHLH136(PRE1 |  | | |
|  |  |  |  | | |  | | | bHLH149 |  | | |
|  |  |  |  | | |  | | | bHLH- BIM1 |  | | |
| nuclear transcription factor Y subunit | | |  | | | NF-Y A-7 | | | NF-Y A2 | NF-YA5 | | |
|  |  |  |  | | | NF-Y A-9 | | | NF-YA9 | NF-YB1 | | |
|  |  |  |  | | | NF-Y B-2 | | | NF-YB4 | NF-YB10 | | |
|  |  |  |  | | | NF-Y B-4 | | | NF-YB8 | NF-YC3 | | |
|  |  |  |  | | | NF-Y C-4 | | | NF-YC6 |  | | |
| bZIP transcription factor | | | RF2b | | | bZIP53 (At3g62420) | | | bZIP16 | bZIP 60 | | |
|  |  |  | bZIP 51 (VIP1) | | | TGA4 (bZIP57) | | | bZIP19 | BZIP61 | | |
|  |  |  |  | | |  | | | bZIP21 (AT1G08320) |  | | |
|  |  |  |  | | |  | | | TGA3 (bZIP22) |  | | |
|  |  |  |  | | |  | | | bZIP30(AT2G21230) |  | | |
|  |  |  |  | | |  | | | BZIP34 |  | | |
|  |  |  |  | | |  | | | bZIP 56 (HY5) |  | | |
|  |  |  |  | | |  | | | TGA4 (bZIP57) |  | | |
|  |  |  |  | | |  | | | BZIP61 |  | | |
|  |  |  |  | | |  | | | TGA1(bZIP65) |  | | |
|  |  |  |  | | |  | | | bZIP68 |  | | |
|  |  |  |  | | |  | | | ASML3 (AT5G07160) |  | | |
|  |  |  |  | | |  | | | AT2G40620 |  | | |
|  |  |  |  | | |  | | | AT1G58110 |  | | |
|  |  |  |  | | |  | | | HYH (AT3G17609) |  | | |
| GATA | | |  | | | GATA 6 | | | GATA 1 | GATA 5 | | |
|  |  |  |  | | | GATA 16 | | | GATA 3 | GATA 15 | | |
|  |  |  |  | | | GATA 20 | | | GATA4 | GATA 17 | | |
|  |  |  |  | | |  | | | GATA5 | GATA 24 | | |
|  |  |  |  | | |  | | | GATA10 |  | | |
|  |  |  |  | | |  | | | GATA15 |  | | |
|  |  |  |  | | |  | | | GATA21 |  | | |
|  |  |  |  | | |  | | | GATA24/ ZML1 |  | | |
| heat stress TF | | | HSFB A-4d | | | HSFB A-2b | | | HSFB A-7a |  | | |
|  |  |  | HSFB C-1b | | | HSFB A-2c | | | HSFB A-1b |  | | |
|  |  |  | HSFB C-2a | | | HSFB A-2e | | | HSFB A-6b |  | | |
|  |  |  |  | | | HSFB B-2a | | | HSFB C-1 |  | | |
|  |  |  |  | | | HSFB B-1 | | | HSFB A-1e |  | | |
|  |  |  |  | | | HSFB B-2a | | |  |  | | |
| **Supplementary Table S2**: Uncommon up- and down-regulated TFs in Rice and Arabidopsis | | | | | | | | | | | | |
| TF Family | | Rice up | | | Rice down | | Arabidopsis up | | | | Arabidopsis down | |
| WRKY | |  | | |  | | WRKY3 | | | | WRKY7 | |
|  |  |  | | |  | | WRKY18 | | | |  | |
|  |  |  | | |  | | WRKY21 | | | |  | |
|  |  |  | | |  | | WRKY26 | | | |  | |
|  |  |  | | |  | | WRKY27 | | | |  | |
|  |  |  | | |  | | WRKY28 | | | |  | |
|  |  |  | | |  | | WRKY30 | | | |  | |
|  |  |  | | |  | | WRKY31 | | | |  | |
|  |  |  | | |  | | WRKY54 | | | |  | |
|  |  |  | | |  | | WRKY58 | | | |  | |
|  |  |  | | |  | | WRKY70 | | | |  | |
|  |  |  | | |  | | WRKY74 | | | |  | |
| NAC | |  | | | NAC 25 | | NAC2 | | | | NAC6 | |
|  |  |  | | |  | | NAC3 | | | |  | |
|  |  |  | | |  | | NAC005 | | | |  | |
|  |  |  | | |  | | NAC010 | | | |  | |
|  |  |  | | |  | | NAC13 | | | |  | |
|  |  |  | | |  | | NAC024 | | | |  | |
|  |  |  | | |  | | NAC025 | | | |  | |
|  |  |  | | |  | | NAC032 | | | |  | |
|  |  |  | | |  | | NAC041 | | | |  | |
|  |  |  | | |  | | NAC047 | | | |  | |
|  |  |  | | |  | | NAC048 | | | |  | |
|  |  |  | | |  | | NAC050 | | | |  | |
|  |  |  | | |  | | NAC052 | | | |  | |
|  |  |  | | |  | | NAC071 | | | |  | |
|  |  |  | | |  | | \| NAC075 \| \| --- \| | | | |  | |
|  |  |  | | |  | | NAC077 | | | |  | |
|  |  |  | | |  | | AC084 | | | |  | |
|  |  |  | | |  | | ANAC087 | | | |  | |
|  |  |  | | |  | | NAC090 | | | |  | |
|  |  |  | | |  | | NAC095 | | | |  | |
|  |  |  | | |  | | NAC103 | | | |  | |
|  |  |  | | |  | | ATAF-like NAC-domain TF | | | |  | |
| MADS-box | | MADS 13, Maf | | | MADS 1 | | SEPALLATA 1 | | | | MADS- SEPALLATA3 | |
|  |  | MADS 16 | | | MADS4 | | AGAMOUS | | | | MYB75 | |
|  |  | MADS 21 | | | MADS7 | | AT1G72350 | | | | LOF2 | |
|  |  | MADS 26 | | | MADS15 | | AT3G05860 | | | |  | |
|  |  | MADS 30 | | | MADS22 | | PHE1///AGL38 | | | |  | |
|  |  | MADS 31 | | | MADS15 | | AGL72 (AT5G51860) | | | |  | |
|  |  | MADS 47 | | | MADS29 | |  | | | |  | |
|  |  | MADS 50 | | |  | |  | | | |  | |
|  |  | MADS 57 | | |  | |  | | | |  | |
| TCP | | PCF2 | | | PCF1 | | TCP2 | | | | TCP13 | |
|  |  | PCF6 | | | PCF5 | | TCP5 | | | |  | |
|  |  | PCF8 | | | TCP7 | | TCP10 | | | |  | |
|  |  | TCP14 | | |  | | TCP17 | | | |  | |
|  |  | TCP15 | | |  | | MEE3 (TCP4 | | | |  | |
| Trihelix TF | | ASR3 | | | ASIL1 | | GT-2 | | | |  | |
|  |  |  | | | GTL1 | | GTL1 | | | |  | |
| G2-like protein | | PCL1 | | | transcription factor LUX | | protein BROTHER OF LUX ARRHYTHMO  LUX  AT3G13040  AT3G04450  AT2G40260 | | | |  | |
| [E2F/DP](http://planttfdb.gao-lab.org/family.php?sp=Ath&fam=E2F/DP) | |  | | | DPB | | DPA  E2F3 | | | |  | |
| APG-like | | APG-like | | |  | |  | | | |  | |
| [C2H2](http://planttfdb.gao-lab.org/family.php?sp=Ath&fam=C2H2) | |  | | |  | | IIIA (AT1G72050) | | | |  | |
| [ZF-HD](http://planttfdb.gao-lab.org/family.php?sp=Ath&fam=ZF-HD) | |  | | |  | | ZFHD1 (AT1G69600) | | | |  | |
