# Supplementary material for: Cold-responsive transcription factors in Arabidopsis and rice: A regulatory network analysis using array data and gene co-expression network
Source: PLoS One. 2023 Jun 8;18(6):e0286324. doi: 10.1371/journal.pone.0286324 (PMC10249815; doi:10.1371/journal.pone.0286324)
Supplement: S3 Table — (DOCX) [file pone.0286324.s003.docx]

| **Supplementary Table S3**: Transcription factor specifications obtained from Plant Transcription Factor Database (<http://planttfdb.gao-lab.org/>) [32] | | | | | | | |
| --- | --- | --- | --- | --- | --- | --- | --- |
| TF Name | Organism  Arabidopsis/Rice | Protein Properties | | | Signature Domain | Number of motifs | Binding motif |
|  |  | Length (aa) | MW (Da) | pI |  |  |  |
| ANT | AT4G37750 | 555 | 61724.9 | 7.8658 | AP2 | 2 | 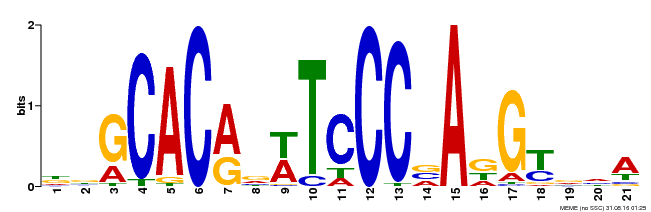 |
|  | Os03g12950 | 642 | 71840.4 | 7.0671 | AP2 | 2 |  |
| ERF 4 | AT3G15210 | 222 | 23740.8 | 9.1569 | AP2/ERF | 1 | 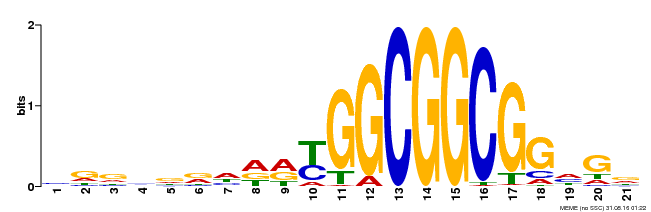 |
|  | Os12g39330 | 402 | 36528.6 | 4.5185 | AP2/ERF | 1 |  |
| ERF 5 | AT5G47230 | 300 | 33809.9 | 4.7948 | AP2/ERF | 1 |  |
|  | Os07g10410.1 | 267 | 29029.5 | 8.395 | AP2/ERF | 1 |  |
| ERF13 | At2g44840 | 226 | 25353.1 | 6.7937 | AP2/ERF | 1 |  |
|  | Os06g11940 | 224 | 24191 | 4.6858 | AP2 | 1 |  |
| ERF38 | AT2G35700 | 194 | 21617.3 | 6.6865 | AP2/ERF | 1 |  |
|  | [Os02g13710.1](http://rice.plantbiology.msu.edu/cgi-bin/ORF_infopage.cgi?orf=LOC_Os02g13710.1) | 248 | 25408.4 | 4.9562 | AP2/ERF | 1 |  |
| ERF73 | AT1G72360 | 260 | 28949.8 | 4.6028 | AP2/ERF | 1 |  |
|  | [Os09g11460.2](http://rice.plantbiology.msu.edu/cgi-bin/ORF_infopage.cgi?orf=LOC_Os09g11460.2) | 233 | 26038.6 | 8.4969 | AP2/ERF | 1 |  |
| ERF74-RAP2-12 | AT1G53910 | 356 | 39656.9 | 4.9538 | AP2/ERF | 1 |  |
|  | [Os05g41780.1](http://rice.plantbiology.msu.edu/cgi-bin/ORF_infopage.cgi?orf=LOC_Os05g41780.1) | 237 | 24113.3 | 9.3484 | AP2/ERF | 1 |  |
| ERF98 | AT3G23230 | 139 | 16096.5 | 7.5189 | AP2/ERF | 1 |  |
|  | [Os02g34260.1](http://rice.plantbiology.msu.edu/cgi-bin/ORF_infopage.cgi?orf=LOC_Os02g34260.1) | 218 | 22448.3 | 11.1567 | AP2/ERF | 1 |  |
| ERF113 | AT5G13330 | 212 | 24287.7 | 9.1153 | AP2/ERF | 1 |  |
|  | [Os06g42990.1](http://rice.plantbiology.msu.edu/cgi-bin/ORF_infopage.cgi?orf=LOC_Os06g42990.1) | 393 | 40886.2 | 9.4028 | AP2/ERF | 1 |  |
| DREB 1A | AT4G25480 | 216 | 24236.1 | 4.8673 | AP2/ERF | 1 | 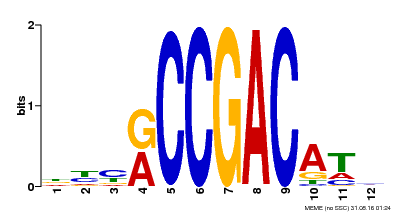 |
|  | [Os09g35030.1](http://rice.plantbiology.msu.edu/cgi-bin/ORF_infopage.cgi?orf=LOC_Os09g35030.1) | 239 | 25390.1 | 4.8433 | AP2/ERF | 1 |  |
| DREB 1B | AT4G25490 | 213 | 23829.6 | 4.7395 | AP2/ERF | 1 |  |
|  | Os09g35010.1 | 219 | 23237.9 | 4.9735 | AP2/ERF | 1 |  |
|  |  |  |  |  |  |  |  |
|  |  |  |  |  |  |  |  |
|  |  |  |  |  |  |  |  |
|  |  |  |  |  |  |  |  |
|  |  |  |  |  |  |  |  |
|  |  |  |  |  |  |  |  |
| **Supplementary Table S3**: Transcription factor specifications obtained from Plant Transcription Factor Database (<http://planttfdb.gao-lab.org/>) [32] | | | | | | | |
| TF Name | Organism  Arabidopsis/Rice | Protein Properties | | | Signature Domain | Number of motifs | Binding motif |
|  |  | Length (aa) | MW (Da) | pI |  |  |  |
| MYB57 | AT3G01530.1 | 206 | 23716.8 | 10.0597 | Myb_DNA-binding | 2 | 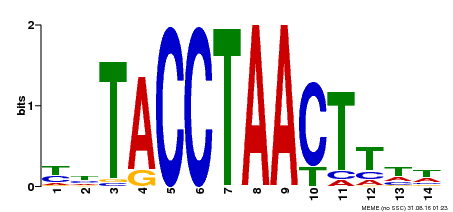 |
|  | Os02g40530.1 | 290 | 32082.8 | 5.2235 | Myb_DNA-binding | 2 |  |
| MYB59 | AT5G59780 | 235 | 27236.7 | 8.6904 | Myb_DNA-binding | 2 | 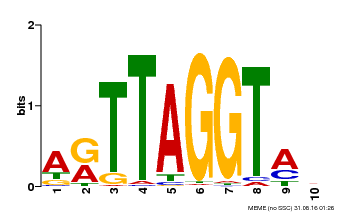 |
|  | [Os01g74410.2](http://rice.plantbiology.msu.edu/cgi-bin/ORF_infopage.cgi?orf=LOC_Os01g74410.2) | 211 | 24075.6 | 6.7855 | Myb_DNA-binding | 1 |  |
| bHLH16/ UNE10b | AT4G00050 | 399 | 43365.2 | 7.5542 | HLH | 1 | 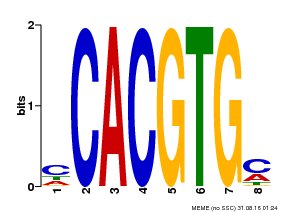 |
|  | Os07g36460.1 | 235 | 25674.9 | 4.834 | HLH | 1 |  |
| bHLH35 | AT5G57150.3 | 219 | 24777.1 | 4.6219 | HLH | 1 |  |
|  | [Os04g23550.2](http://rice.plantbiology.msu.edu/cgi-bin/ORF_infopage.cgi?orf=LOC_Os04g23550.2) | 190 | 20269.2 | 5.9395 | HLH | 1 |  |
| bHLH79 | AT5G62610.1 | 281 | 30569.8 | 6.0652 | HLH | 1 |  |
|  | [Os02g47660.1](http://rice.plantbiology.msu.edu/cgi-bin/ORF_infopage.cgi?orf=LOC_Os02g47660.1) | 362 | 38638.6 | 7.3379 | HLH | 1 |  |
| bHLH128 | AT1G05805.1 | 362 | 38951.2 | 7.5291 | HLH | 1 |  |
|  | [Os07g39940.1](http://rice.plantbiology.msu.edu/cgi-bin/ORF_infopage.cgi?orf=LOC_Os07g39940.1) | 280 | 30453.1 | 6.0914 | HLH | 1 |  |
| bHLH129 | AT2G43140.1 | 297 | 32431.4 | 7.9584 | HLH | 1 |  |
|  | [Os03g10770.1](http://rice.plantbiology.msu.edu/cgi-bin/ORF_infopage.cgi?orf=LOC_Os03g10770.1) | 325 | 34667.5 | 6.2514 | HLH | 1 |  |
| bHLH137 | AT5G50915.2 | 286: | 31773.4 | 6.4033 | HLH | 1 |  |
|  | Os08g42470.1 | 292 | 31606.4 | 6.6036 | HLH | 1 |  |
| bHLH148 | AT3G06590.2 | 221 | 24256 | 12.3375 | HLH | 1 |  |
|  | [Os03g53020.1](http://rice.plantbiology.msu.edu/cgi-bin/ORF_infopage.cgi?orf=LOC_Os03g53020.1) | 300 | 32907.6 | 5.2973 | HLH | 1 |  |
| bHLH 59/ UNE12 | AT4G02590.3 | 247 | 26834.6 | 9.2324 | HLH | 1 |  |
|  | Os02g02480.3 | 237 | 25593 | 4.9237 | HLH | 1 |  |
| bHLH102/BIM2 | AT1G69010 | 311 | 34486.9 | 5.0332 | HLH | 1 |  |
|  | [Os12g41650.5](http://rice.plantbiology.msu.edu/cgi-bin/ORF_infopage.cgi?orf=LOC_Os12g41650.5) | 318 | 33588.6 | 6.7765 | HLH | 1 |  |
| bHLH105/ ILR3 | AT5G54680 | 234 | 25487.9 | 8.8356 | HLH | 1 |  |
|  | Os08g04390.2 | 254 | 27226.8 | 5.8206 | HLH | 1 |  |
| BHLH116/ICE1 | AT3G26744 | 494 | 53538.9 | 5.1164 | HLH | 1 |  |
|  | Os01g50940.1 | 474 | 50668.3 | 8.4746 | HLH | 1 |  |
| NFYA-4 | AT2G34720 | 198 | 22258.7 | 9.2692 | CBFB_NFYA | 1 |  |
|  | Os03g48970.4 | 247 | 26606.5 | 8.7304 | CBFB_NFYA | 1 |  |
| NFYA-10 | AT5G06510 | 220 | 24691 | 9.4736 | CBFB_NFYA | 1 |  |
|  | [Os12g42400.4](http://rice.plantbiology.msu.edu/cgi-bin/ORF_infopage.cgi?orf=LOC_Os12g42400.4) | 206 | 22165.4 | 9.0889 | CBFB_NFYA | 1 |  |

| **Supplementary Table S3**: Transcription factor specifications obtained from Plant Transcription Factor Database (<http://planttfdb.gao-lab.org/>) [32] | | | | | | | | |
| --- | --- | --- | --- | --- | --- | --- | --- | --- |
| TF Name | Organism  Arabidopsis/Rice | Protein Properties | | |  |  |  | |
|  |  | Length (aa) | MW (Da) | pI | Signature Domain | Number of motifs | Binding motif | |
| bZIP20/TGA2 | AT5G06950.4 | 330 | 36684.3 | 9.0124 | bZIP_1 | 1 | 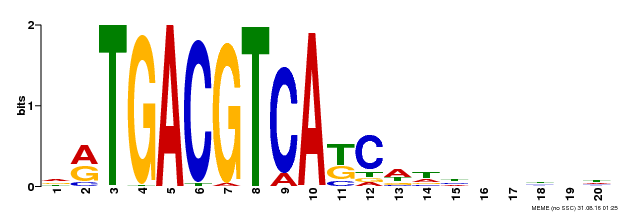 | |
|  | [Os01g59350.2](http://rice.plantbiology.msu.edu/cgi-bin/ORF_infopage.cgi?orf=LOC_Os01g59350.2) | 365 | 39852.5 | 7.8662 | bZIP_1 | 1 |  |  |
| bZIP45/TGA6 | AT3G12250.5 | 303 | 33963.4 | 10.0928 | bZIP_1 | 1 |  |  |
|  | [Os05g49420.1](http://rice.plantbiology.msu.edu/cgi-bin/ORF_infopage.cgi?orf=LOC_Os05g49420.1) | 381 | 40408.5 | 9.6898 | bZIP_1 | 1 |  |  |
| bZIP 60 | AT1G42990.1 | 295 | 33174.1 | 4.3626 | bZIP_1 | 1 |  |  |
|  | [Os07g44950.1](http://rice.plantbiology.msu.edu/cgi-bin/ORF_infopage.cgi?orf=LOC_Os07g44950.1) | 569 | 60071.6 | 5.6826 | bZIP_1 | 1 |  |  |
| GATA 11 | AT1G08010.2 | 303 | 33839.1 | 8.5871 | GATA | 1 | 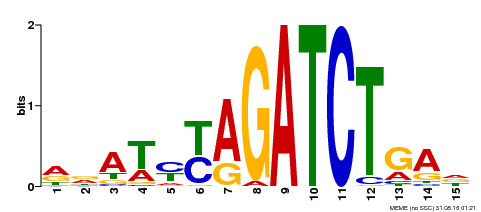 | |
|  | [Os02g12790.1](http://rice.plantbiology.msu.edu/cgi-bin/ORF_infopage.cgi?orf=LOC_Os02g12790.1) | 354 | 37637.9 | 9.7819 | GATA | 1 |  |  |
| GATA 22 | AT4G26150.1 | 352 | 39345.3 | 9.5931 | GATA | 1 |  |  |
|  | Os06g37450.1 | 391 | 41083.7 | 9.8163 | GATA | 1 |  |  |
| GATA 23 | AT5G26930.1 | 120 | 13238.7 | 10.74 | GATA | 1 |  |  |
|  | Os01g24070.2 | 102 | 10989.4 | 9.9907 | GATA | 1 |  |  |
| HSF A-3 | AT5G03720.1 | 412 | 46458.1 | 5.131 | HSF_DNA-bind | 1 | 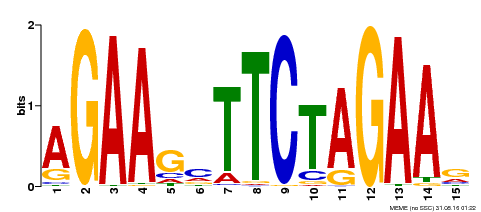 | |
|  | Os02g32590.2 | 451 | 49960 | 4.7012 | HSF_DNA-bind | 1 |  |  |
| HSF A-9 | AT5G54070.1 | 331 | 38146.2 | 5.373 | HSF_DNA-bind | 1 |  |  |
|  | Os03g12370.3 | 407 | 45134.4 | 5.0466 | HSF_DNA-bind | 1 |  |  |
| HSF B-2b | AT4G11660.1 | 377 | 39705.1 | 4.6287 | HSF_DNA-bind | 1 |  |  |
|  | Os08g43334.2 | 391 | 41374.1 | 5.0078 | HSF_DNA-bind | 1 |  |  |
| HSF B4 | AT1G46264.1 | 348 | 39615.4 | 7.8846 | HSF_DNA-bind | 1 |  |  |
|  | Os07g44690.1 | 311 | 34450.8 | 7.0637 | HSF_DNA-bind | 1 |  |  |
| WRKY1/ZAP1 | AT2G04880.2 | 463 | 51209.9 | 7.0213 | WRKY | 2 | 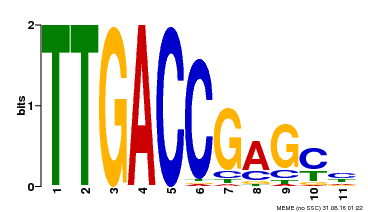 |  |
|  | Os01g14440.1 | 584 | 60502.4 | 8.7383 | WRKY | 1 |  |  |

| **Supplementary Table S3**: Transcription factor specifications obtained from Plant Transcription Factor Database (<http://planttfdb.gao-lab.org/>) [32] | | | | | | | |
| --- | --- | --- | --- | --- | --- | --- | --- |
| TF Name | Organism | Protein Properties | | | Signature Domain | Number of motifs | Binding motif |
|  | Arabidopsis/Rice | Length (aa) | MW (Da) | pI |  |  |  |
| PLT2 | At1g51190 | 568 | 62233.1 | 6.8282 | AP2 | 2 |  |
|  | Os06g44750.1 | 264 | 28123.8 | 9.3971 | AP2 | 1 |  |
| ERF39 | AT4G16750 | 179 | 19909.1 | 6.6896 | AP2 | 1 | 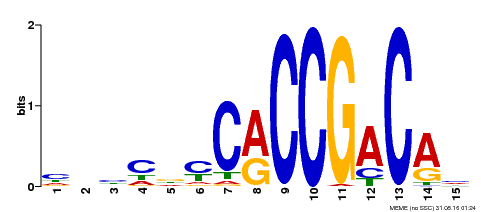 |
|  | [Os01g10370.1](http://rice.plantbiology.msu.edu/cgi-bin/ORF_infopage.cgi?orf=LOC_Os01g10370.1) | 229 | 24558.4 | 6.419 | AP2 | 1 |  |
| ERF54 | AT4G28140 | 292 | 33403.2 | 5.431 | AP2 | 1 | 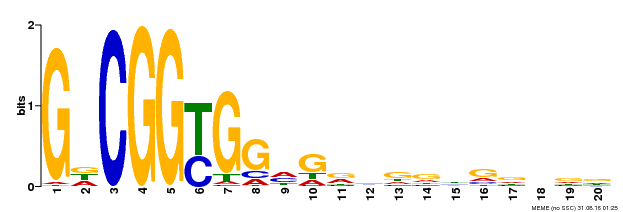 |
|  | [Os01g46870.1](http://rice.plantbiology.msu.edu/cgi-bin/ORF_infopage.cgi?orf=LOC_Os01g46870.1) | 300 | 31538.7 | 4.2163 | AP2 | 1 | 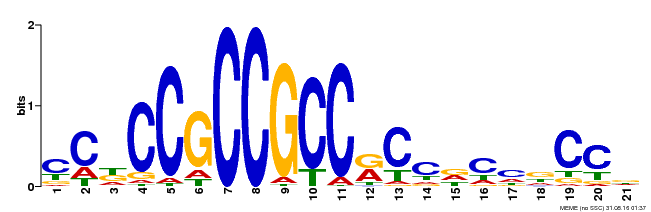 |
| MYB5 | AT3G13540 | 249 | 27793.5 | 8.285 | Myb_DNA-binding | 2 |  |
|  | Os05g41166.1 | 395 | 45133.3 | 5.0147 |  | 2 |  |
| MYB37/RAX1 | AT5G23000 | 329 | 37045.4 | 8.6791 | Myb_DNA-binding | 2 |  |
|  | Os01g09590.1 | 265 | 27979.3 | 6.0167 |  | 2 |  |
| MYB38/RAX2 | AT2G36890 | 298 | 34057.1 | 7.1008 |  | 2 |  |
|  | Os02g42870.1 | 301 | 32154.3 | 7.4607 | Myb_DNA-binding | 2 | 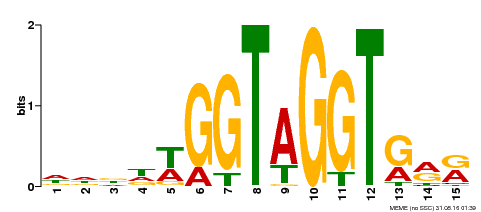 |

| Supplementary Table S3: Transcription factor specifications obtained from Plant Transcription Factor Database (<http://planttfdb.gao-lab.org/>) [32] | | | | | | | |
| --- | --- | --- | --- | --- | --- | --- | --- |
| TF Name | Organism | Protein Properties | | | Signature Domain | Number of motifs | Binding motif |
|  |  | Length (aa) | MW (Da) | pI |  |  |  |
| MYB44 | AT5G67300 | 305 | 33268.2 | 8.1123 | Myb_DNA-binding | 2 | 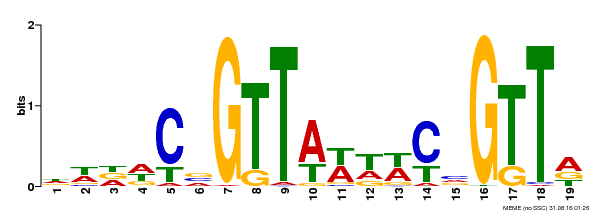 |
|  | Os01g52410.1 | 299 | 31761.2 | 4.6593 | Myb_DNA-binding | 2 |  |
| MYB84/RAX3 | AT3G49690 | 310 | 35578.1 | 7.311 | Myb_DNA-binding | 2 | 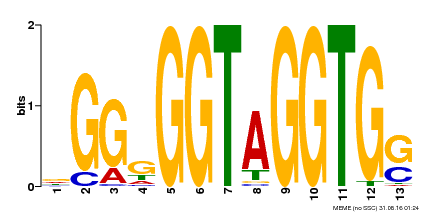 |
|  | Os09g01960.1 | 320 | 34573.3 | 10.2107 | Myb_DNA-binding | 2 | 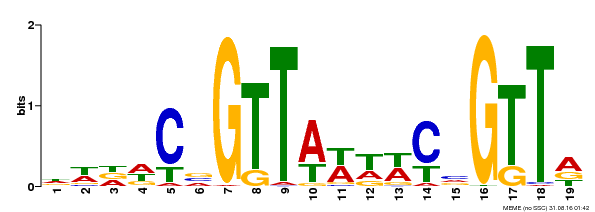 |
| bHLH112 | AT1G61660 | 371 | 40502.3 | 6.0906 | HLH | 1 |  |
|  | Os04g53990.1 | 438 | 45811.3 | 7.5731 | HLH | 1 |  |
| bHLH113 | AT3G19500 | 270 | 28787 | 5.0095 | HLH | 1 |  |
|  | Os03g55220.2 | 318 | 33168.3 | 7.8588 | HLH | 1 |  |
|  |  |  |  |  |  |  |  |
|  |  |  |  |  |  |  |  |

| **Supplementary Table S3**: Transcription factor specifications obtained from Plant Transcription Factor Database (<http://planttfdb.gao-lab.org/>) [32] | | | | | | | |
| --- | --- | --- | --- | --- | --- | --- | --- |
| TF Name | Organism | Protein Properties | | | Signature Domain | Number of motifs | Binding motif |
|  |  | Length (aa) | MW (Da) | pI |  |  |  |
| NF-Y B-3 | AT4G14540 | 161 | 17186 | 6.1171 | NF-YB | 1 |  |
|  | Os05g49780.1 | 142 | 15300 | 4.732 | NF-YB | 1 |  |
| NF-Y B-4 | AT1G09030 | 139 | 15741.4 | 7.5093 | NF-YB | 1 | 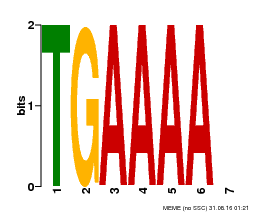 |
|  | Os05g38820.4 | 144 | 15147 | 5.2724 | NF-YB | 1 |  |
| NF-Y B-9 | AT1G21970 | 238 | 26070 | 5.9417 | NF-YB | 1 |  |
|  | Os06g17480.1 | 251 | 25965.1 | 5.5502 | NF-YB | 1 |  |
| NF-Y C-2 | AT1G56170 | 199 | 23124 | 6.0246 | NF-YC | 1 |  |
|  | Os03g14669.2 | 247 | 25896.1 | 5.0564 | NF-YC | 1 |  |
| bZIP17 | AT2G40950 | 721 | 78437.9 | 6.2704 | bZIP-1 | 1 |  |
|  | Os02g10140.1 | 304 | 31720.6 | 9.755 | bZIP-1 | 1 |  |
| TCP21 | AT5G08330 | 239 | 24751.6 | 10.2008 | TCP | 1 | 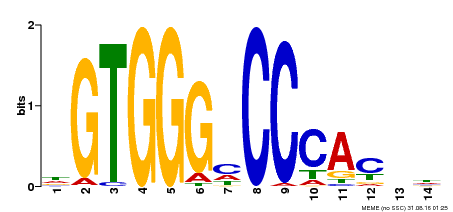 |
|  | Os07g05720.1 | 446 | 47256.3 | 9.4979 | TCP | 1 | 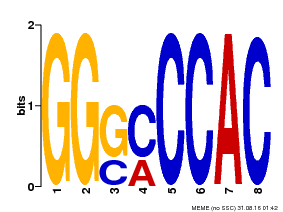 |
