# Supplementary material for: Cold-responsive transcription factors in Arabidopsis and rice: A regulatory network analysis using array data and gene co-expression network
Source: PLoS One. 2023 Jun 8;18(6):e0286324. doi: 10.1371/journal.pone.0286324 (PMC10249815; doi:10.1371/journal.pone.0286324)
Supplement: S4 Table — (DOCX) [file pone.0286324.s004.docx]

| **Supplementary Table S4**: Protein BLAST [33] results of common up- and down-regulated TF genes in Arabidopsis and Rice. | | | | | | | | | | | | | | | |
| --- | --- | --- | --- | --- | --- | --- | --- | --- | --- | --- | --- | --- | --- | --- | --- |
| No. | **Up-regulated** | Max score | Total score | Query cover | E value | Percent identity | Accession length | No. | **Down-regulated** | Max score | Total score | Query cover | E value | Percent identity | Accession length |
| 1 | ANT | 407 | 472 | 86% | 7e-140 | 57.35% | 642 | 1 | PLT2 | 199 | 265 | 35% | 2e-64 | 83.78% | 263 |
| 2 | ERF 4 | 84.0 | 84.0 | 25% | 2e-23 | 68.42% | 402 | 2 | ERF39 | 126 | 126 | 41% | 2e-41 | 80.00% | 228 |
| 3 | ERF 5 | 73.2 | 73.2 | 22% | 1e-19 | 54.41% | 266 | 3 | ERF54 | 81.3 | 81.3 | 21% | 2e-22 | 60.32% | 299 |
| 4 | ERF13 | 63.9 | 63.9 | 32% | 4e-17 | 50.65% | 223 | 4 | MYB5 | 147 | 147 | 60% | 1e-46 | 47.33% | 394 |
| 5 | ERF38 | 114 | 114 | 41% | 2e-36 | 73.75% | 247 | 5 | MYB37/RAX1 | 187 | 187 | 33% | 1e-62 | 72.07% | 264 |
| 6 | ERF73 | 96.3 | 96.3 | 21% | 1e-28 | 75.00% | 232 | 6 | MYB38/RAX2 | 166 | 166 | 39% | 2e-54 | 64.10% | 300 |
| 7 | ERF74-RAP2-12 | 72.0 | 72.0 | 17% | 4e-19 | 69.35% | 236 | 7 | MYB44 | 102 | 102 | 33% | 5e-30 | 48.54% | 298 |
| 8 | ERF98 | 55.5 | 55.5 | 51% | 6e-15 | 57.89% | 217 | 8 | MYB84/RAX3 | 100 | 130 | 33% | 6e-29 | 47.57% | 319 |
| 9 | ERF113 | 76.3 | 76.3 | 32% | 7e-21 | 59.15% | 392 | 9 | bHLH112 | 127 | 127 | 21% | 1e-37 | 78.67% | 437 |
| 10 | DREB 1A | 129 | 129 | 87% | 1e-41 | 42.33% | 238 | 10 | bHLH113 | 119 | 119 | 38% | 1e-36 | 55.08% | 317 |
| 11 | DREB 1B | 93.2 | 93.2 | 88% | 3e-28 | 45.97% | 218 | 11 | NF-Y B-3 | 162 | 162 | 56% | 7e-57 | 78.02% | 141 |
| 12 | MYB57 | 161 | 161 | 51% | 8e-54 | 66.36% | 289 | 12 | NF-Y B-4 | 131 | 131 | 74% | 3e-45 | 53.40% | 143 |
| 13 | MYB59 | 130 | 130 | 33% | 3e-42 | 70.89% | 210 | 13 | NF-Y B-9 | 162 | 162 | 42% | 3e-54 | 71.84% | 250 |
| 14 | bHLH16/ UNE10b | 29.6 | 29.6 | 13% | 5e-05 | 34.62% | 234 | 14 | NF-Y C-2 | 180 | 180 | 51% | 5e-62 | 80.00% | 246 |
| 15 | bHLH35 | 97.4 | 97.4 | 60% | 4e-30 | 37.97% | 189 | 15 | bZIP17 | 33.9 | 33.9 | 4% | 6e-06 | 53.33% | 303 |
| 16 | bHLH79 | 129 | 129 | 27% | 1e-39 | 75.00% | 361 | 16 | TCP21 | 43.1 | 43.1 | 27% | 3e-09 | 37.14% | 445 |
| 17 | BHLH116/ICE1 | 71.2 | 71.2 | 10% | 2e-17 | 60.38% | 473 |  |  |  |  |  |  |  |  |
| 18 | bHLH128 | 63.2 | 63.2 | 18% | 8e-16 | 50.00% | 279 |  |  |  |  |  |  |  |  |
| 19 | bHLH129 | 47.8 | 47.8 | 18% | 9e-11 | 43.64% | 324 |  |  |  |  |  |  |  |  |
| 20 | bHLH137 | 184 | 184 | 54% | 1e-61 | 57.40% | 291 |  |  |  |  |  |  |  |  |
| 21 | bHLH148 | - | - | - | - | - | - |  |  |  |  |  |  |  |  |
| 22 | bHLH 59/ UNE12 | - | - | - | - | - | - |  |  |  |  |  |  |  |  |
| 23 | bHLH102/BIM2 | 36.2 | 36.2 | 17% | 4e-07 | 33.96% | 317 |  |  |  |  |  |  |  |  |
| 24 | bHLH105/ ILR3 | 217 | 217 | 83% | 1e-75 | 59.31% | 253 |  |  |  |  |  |  |  |  |
| 25 | NFYA-4 | 123 | 123 | 62% | 8e-40 | 51.59% | 246 |  |  |  |  |  |  |  |  |
| 26 | NFYA-10 | 79.7 | 79.7 | 31% | 1e-22 | 45.35% | 205 |  |  |  |  |  |  |  |  |
| 27 | bZIP20/TGA2 | 293 | 293 | 60% | 4e-102 | 72.28% | 364 |  |  |  |  |  |  |  |  |
| 28 | bZIP45/TGA6 | 33.1 | 33.1 | 16% | 6e-06 | 39.62% | 380 |  |  |  |  |  |  |  |  |
| 29 | bZIP 60 | 57.0 | 57.0 | 20% | 2e-13 | 45.00% | 568 |  |  |  |  |  |  |  |  |
| 30 | GATA 11 | 62.0 | 62.0 | 10% | 2e-15 | 69.70% | 353 |  |  |  |  |  |  |  |  |
| 31 | GATA 22 | 95.1 | 95.1 | 15% | 3e-26 | 74.55% | 390 |  |  |  |  |  |  |  |  |
| 32 | GATA 23 | 60.1 | 60.1 | 66% | 5e-18 | 41.05% | 101 |  |  |  |  |  |  |  |  |
| 33 | HSF A-3 | 229 | 229 | 70% | 5e-75 | 43.87% | 450 |  |  |  |  |  |  |  |  |
| 34 | HSF A-9 | 148 | 148 | 53% | 7e-46 | 39.90% | 406 |  |  |  |  |  |  |  |  |
| 35 | HSF B-2b | 238 | 238 | 83% | 7e-80 | 46.04% | 390 |  |  |  |  |  |  |  |  |
| 36 | HSF B4 | 249 | 249 | 85% | 1e-85 | 50.49% | 310 |  |  |  |  |  |  |  |  |
| 37 | WRKY1/ZAP1 | 100 | 171 | 29% | 5e-27 | 52.63% | 583 |  |  |  |  |  |  |  |  |
