# Supplementary material for: Cold-responsive transcription factors in Arabidopsis and rice: A regulatory network analysis using array data and gene co-expression network
Source: PLoS One. 2023 Jun 8;18(6):e0286324. doi: 10.1371/journal.pone.0286324 (PMC10249815; doi:10.1371/journal.pone.0286324)
Supplement: S5 Table — The metabolic pathways of each interacted protein were obtained from KEGG Pathway database [50]. (DOCX) [file pone.0286324.s005.docx]

| **Supplementary Table S5**: TF interactions with other TFs or proteins were obtained from CORNET [34] based on IntACt, TAIR and AtPID databases. The metabolic pathways of each interacted protein were obtained from KEGG Pathway database [50]. | | | | |
| --- | --- | --- | --- | --- |
| TF name | Interacted protein 2 | Protein name | Metabolic pathway | |
| ERF 4 | AT2G45640 | histone deacetylase complex subunit SAP18 | | \| [ath03013](https://www.genome.jp/pathway/ath03013+AT2G45640) \| Nucleocytoplasmic transport \| \| --- \| --- \|  \| [ath03015](https://www.genome.jp/pathway/ath03015+AT2G45640) \| mRNA surveillance pathway \| \| --- \| --- \| |
|  | AT1G64280 | NPR1 | | [ath04075](https://www.genome.jp/pathway/ath04075+AT1G19350)  Plant hormone signal transduction |
|  | AT3G47640 | PYE; basic helix-loop-helix (bHLH) DNA-binding superfamily protein | |  |
| ERF73 | AT2G38490 | CIPK22; CBL-interacting Serine/Threonine-kinase | |  |
| ERF74-RAP2-12 | AT2G44040 | 4-hydroxy-tetrahydrodipicolinate reductase [EC:1.17.1.8] \| (RefSeq) Dihydrodipicolinate reductase, bacterial/plant | | \| [ath00261](https://www.genome.jp/pathway/ath00261+AT2G44040) \| Monobactam biosynthesis \| \| --- \| --- \|  \| [ath00300](https://www.genome.jp/pathway/ath00300+AT2G44040) \| Lysine biosynthesis \| \| --- \| --- \|  \| [ath01100](https://www.genome.jp/pathway/ath01100+AT2G44040) \| Metabolic pathways \| \| --- \| --- \|  \| [ath01110](https://www.genome.jp/pathway/ath01110+AT2G44040) \| Biosynthesis of secondary metabolites \| \| --- \| --- \|  \| [ath01230](https://www.genome.jp/pathway/ath01230+AT2G44040) \| Biosynthesis of amino acids \| \| --- \| --- \| |
| ERF113 | AT1G64280 | regulatory protein NPR1 \| (RefSeq) NPR1; regulatory protein (NPR1) | |  |
|  | AT1G20610 | G2/mitotic-specific cyclin-B, other \| (RefSeq) CYCB2;3; Cyclin B2;3 | |  |
| DREB 1A | AT1G64280 | regulatory protein NPR1 \| (RefSeq) NPR1; regulatory protein (NPR1) | |  |
|  | AT5G51990 | CBF4; C-repeat-binding factor 4 | |  |
| DREB 1B/CBF1 | AT1G19350 | brassinosteroid resistant 1/2 \| (RefSeq) BES1; Brassinosteroid signaling positive regulator (BZR1) family protein | | [ath04075](https://www.genome.jp/pathway/ath04075+AT1G19350)  Plant hormone signal transduction |
|  | AT4G16420 | transcriptional adapter 2-alpha \| (RefSeq) ADA2B; ADA2 2B | |  |
|  | AT3G07740 | transcriptional adapter 2-alpha \| (RefSeq) ADA2A; ADA2 2A | |  |
|  | AT4G25480 | EREBP-like factor \| (RefSeq) DREB1A; dehydration response element B1A | |  |
|  | AT5G51990 | CBF4; C-repeat-binding factor 4 | |  |
|  | AT1G64280 | regulatory protein NPR1 \| (RefSeq) NPR1; regulatory protein (NPR1) | |  |
|  | AT1G19350 | brassinosteroid resistant 1/2 \| (RefSeq) BES1; Brassinosteroid signaling positive regulator (BZR1) family protein | | \| [ath04075](https://www.genome.jp/pathway/ath04075+AT1G19350) \| Plant hormone signal transduction \| \| --- \| --- \| |
| MYB57 | AT3G27810 | transcription factor MYB, plant \| (RefSeq) MYB21; myb domain protein 21 | |  |
|  | AT3G53200 | transcription factor MYB, plant \| (RefSeq) MYB27; myb domain protein 27 | |  |
|  | AT3G09890 | Ankyrin repeat family protein | |  |
| MYB59 | AT5G40350 | transcription factor MYB, plant \| (RefSeq) MYB24; myb domain protein 24 | |  |
|  | AT3G46130 | transcription factor MYB, plant \| (RefSeq) MYB48; myb domain protein 48 | |  |
|  | AT4G24470 | ZIM; GATA-type zinc finger protein with TIFY domain-containing protein | |  |
|  | AT1G54330 | NAC020; NAC domain containing protein 20 | |  |
|  | AT1G64280 | egulatory protein NPR1 \| (RefSeq) NPR1; regulatory protein (NPR1) | |  |

| **Supplementary Table S5**: TF interactions with other TFs or proteins were obtained from CORNET [34] based on IntACt, TAIR and AtPID databases. The metabolic pathways of each interacted protein were obtained from KEGG Pathway database [50]. | | | |
| --- | --- | --- | --- |
| TF name | Interacted protein 2 | Protein name | Metabolic pathway |
| bHLH16/ UNE10 | AT1G32230 | RCD1; WWE protein-protein interaction domain protein family |  |
|  | AT1G64280 | egulatory protein NPR1 \| (RefSeq) NPR1; regulatory protein (NPR1) |  |
| bHLH 59/ UNE12 | AT1G03040 | basic helix-loop-helix (bHLH) DNA-binding superfamily protein |  |
|  | AT4G30980 | LRL2; LJRHL1-like 2 |  |
|  | AT1G61660 | basic helix-loop-helix (bHLH) DNA-binding superfamily protein |  |
|  | AT3G47640 | PYE; basic helix-loop-helix (bHLH) DNA-binding superfamily protein |  |
|  | AT2G34040 | Apoptosis inhibitory protein 5 (API5) |  |
|  | AT1G31050 | basic helix-loop-helix (bHLH) DNA-binding superfamily protein |  |
|  | AT3G27420 | bromodomain testis-specific protein |  |
|  | AT3G12930 | Lojap-related protein |  |
|  | AT2G42870 | PAR1; phy rapidly regulated 1 |  |
|  | AT4G38230 | calcium-dependent protein kinase [EC:2.7.11.1] \| (RefSeq) CPK26; calcium-dependent protein kinase 26 | \| [ath04075](https://www.genome.jp/pathway/ath04075+AT1G19350) \| Plant hormone signal transduction \| \| --- \| --- \| |
|  | AT4G25315 | Expressed protein |  |
|  | AT1G64620 | Dof-type zinc finger DNA-binding family protein |  |
|  | AT3G47680 | DNA binding protein |  |
|  | AT4G24840 | conserved oligomeric Golgi complex subunit 2 \| (RefSeq) oligomeric golgi complex subunit-like protein |  |
|  | AT5G14070 | ROXY2; Thioredoxin superfamily protein |  |
|  | AT3G28715 | V-type H+-transporting ATPase subunit d \| (RefSeq) ATPase, V0/A0 complex, subunit C/D | \| [ath00190](https://www.genome.jp/pathway/ath00190+AT3G28715) \| Oxidative phosphorylation \| \| --- \| --- \|  \| [ath01100](https://www.genome.jp/pathway/ath01100+AT3G28715) \| Metabolic pathways \| \| --- \| --- \|  \| [ath04145](https://www.genome.jp/pathway/ath04145+AT3G28715) \| Phagosome \| \| --- \| --- \| |
|  | AT1G22630 | SSUH2-like protein |  |
|  | AT5G03500 | mediator of RNA polymerase II transcription subunit 7 \| (RefSeq) Mediator complex, subunit Med7 |  |
|  | AT1G62990 | KNAT7; homeobox knotted-like protein |  |
| bHLH 59/ UNE12 | AT2G24860 | DnaJ/Hsp40 cysteine-rich domain superfamily protein |  |
|  | AT5G11980 | conserved oligomeric Golgi complex subunit 8 \| (RefSeq) conserved oligomeric Golgi complex component-related / COG complex component-like protein |  |
|  | AT5G15802 | chaperone |  |
|  | AT2G24860 | DnaJ/Hsp40 cysteine-rich domain superfamily protein |  |
|  | AT5G45620 | proteasome regulatory subunit N9 \| (RefSeq) Proteasome component (PCI) domain protein | \| [ath03050](https://www.genome.jp/pathway/ath03050+AT5G45620) \| Proteasome \| \| --- \| --- \| |
|  | AT1G78680 | gamma-glutamyl hydrolase [EC:3.4.19.9] \| (RefSeq) GGH2; gamma-glutamyl hydrolase 2 | \| [ath00790](https://www.genome.jp/pathway/ath00790+AT1G78680) \| Folate biosynthesis \| \| --- \| --- \|  \| [ath01240](https://www.genome.jp/pathway/ath01240+AT1G78680) \| Biosynthesis of cofactors \| \| --- \| --- \| |
|  | AT5G15330 | SPX4; SPX domain-containing protein 4 |  |
|  | AT2G24020 | Putative BCR, YbaB family COG0718 |  |
|  | AT3G06720 | importin subunit alpha-6/7 \| (RefSeq) IMPA-1; importin alpha isoform 1 | \| [ath03013](https://www.genome.jp/pathway/ath03013+AT3G06720) \| Nucleocytoplasmic transport \| \| --- \| --- \| |
| bHLH 59/ UNE12 | AT2G24860 | DnaJ/Hsp40 cysteine-rich domain superfamily protein |  |
|  | AT5G11980 | conserved oligomeric Golgi complex subunit 8 \| (RefSeq) conserved oligomeric Golgi complex component-related / COG complex component-like protein |  |
|  | AT5G15802 | chaperone |  |
|  | AT2G24860 | DnaJ/Hsp40 cysteine-rich domain superfamily protein |  |
|  | AT5G45620 | proteasome regulatory subunit N9 \| (RefSeq) Proteasome component (PCI) domain protein | \| [ath03050](https://www.genome.jp/pathway/ath03050+AT5G45620) \| Proteasome \| \| --- \| --- \| |
|  | AT1G78680 | gamma-glutamyl hydrolase [EC:3.4.19.9] \| (RefSeq) GGH2; gamma-glutamyl hydrolase 2 | \| [ath00790](https://www.genome.jp/pathway/ath00790+AT1G78680) \| Folate biosynthesis \| \| --- \| --- \|  \| [ath01240](https://www.genome.jp/pathway/ath01240+AT1G78680) \| Biosynthesis of cofactors \| \| --- \| --- \| |
|  | AT5G15330 | SPX4; SPX domain-containing protein 4 |  |

| **Supplementary Table S5**: TF interactions with other TFs or proteins were obtained from CORNET [34] based on IntACt, TAIR and AtPID databases. The metabolic pathways of each interacted protein were obtained from KEGG Pathway database [50]. | | | |
| --- | --- | --- | --- |
| TF name | Interacted protein 2 | Protein name | Metabolic pathway |
| bHLH 59/ UNE12 | AT2G24020 | Putative BCR, YbaB family COG0718 |  |
|  | AT3G06720 | importin subunit alpha-6/7 \| (RefSeq) IMPA-1; importin alpha isoform 1 | \| [ath03013](https://www.genome.jp/pathway/ath03013+AT3G06720) \| Nucleocytoplasmic transport \| \| --- \| --- \| |
| bHLH102/BIM2 | AT1G19350 | brassinosteroid resistant 1/2 \| (RefSeq) BES1; Brassinosteroid signaling positive regulator (BZR1) family protein | [ath04075](https://www.genome.jp/pathway/ath04075+AT1G19350)  Plant hormone signal transduction |
|  | AT1G64280 | regulatory protein NPR1 \| (RefSeq) NPR1; regulatory protein (NPR1) |  |
| bHLH105/ ILR3 | AT1G62990 | KNAT7; homeobox knotted-like protein |  |
|  | AT1G32230 | RCD1; WWE protein-protein interaction domain protein family |  |
| bHLH128 | AT2G27970 | cyclin-dependent kinase regulatory subunit CKS1 \| (RefSeq) CKS2; CDK-subunit 2 |  |
|  | AT3G11520 | G2/mitotic-specific cyclin-B, other \| (RefSeq) CYCB1;3; CYCLIN B1;3 |  |
| bHLH129 | AT3G11520 | G2/mitotic-specific cyclin-B, other \| (RefSeq) CYCB1;3; CYCLIN B1;3 |  |
|  | AT3G50630 | KRP2; KIP-related protein 2 |  |
|  | AT2G27970 | cyclin-dependent kinase regulatory subunit CKS1 \| (RefSeq) CKS2; CDK-subunit 2 |  |
| bHLH148 | AT1G09250 | basic helix-loop-helix (bHLH) DNA-binding superfamily protein |  |
|  | AT4G36540 | BEE2; BR enhanced expression 2 |  |
|  | AT3G49580 | LSU1; response to low sulfur 1 |  |
|  | AT5G15160 | BNQ2; BANQUO 2 |  |
|  | AT1G59640 | BPEp; transcription factor BIG PETAL P (BPE) |  |
|  | AT1G64280 | regulatory protein NPR1 \| (RefSeq) NPR1; regulatory protein (NPR1) |  |
| NFYA-4 | AT4G34530 | CIB1; cryptochrome-interacting basic-helix-loop-helix 1 |  |
|  | AT4G14540 | nuclear transcription Y subunit beta \| (RefSeq) NF-YB3; nuclear factor Y, subunit B3 |  |
|  | AT1G30500 | nuclear transcription factor Y, alpha \| (RefSeq) NF-YA7; nuclear factor Y, subunit A7 |  |
|  | AT4G19700 | E3 ubiquitin-protein ligase BOI and related proteins [EC:2.3.2.27] \| (RefSeq) RING; SBP (S-ribonuclease binding protein) family protein |  |
|  | AT3G08530 | clathrin heavy chain \| (RefSeq) Clathrin, heavy chain |  |
|  | AT3G49580 | clathrin heavy chain | \| [ath04144](https://www.genome.jp/pathway/ath04144+AT3G08530) \| Endocytosis \| \| --- \| --- \| |
|  | AT2G19650 | Cysteine/Histidine-rich C1 domain family protein |  |
| NFYA-10 | AT3G05690 | nuclear transcription factor Y, alpha \| (RefSeq) NF-YA2; nuclear factor Y, subunit A2 |  |
| bZIP20/TGA2 | AT1G64280 | regulatory protein NPR1 \| (RefSeq) NPR1; regulatory protein (NPR1) |  |
|  | AT4G19660 | regulatory protein NPR1 \| (RefSeq) NPR1; regulatory protein (NPR1) |  |
|  | AT1G28480 | GRX480; Thioredoxin superfamily protein |  |
|  | AT1G02450 | NIMIN1; NIM1-interacting 1 |  |
|  | AT5G45110 | regulatory protein NPR1 \| (RefSeq) NPR3; NPR1-like protein 3 |  |
|  | AT3G12250 | transcription factor TGA \| (RefSeq) TGA6; TGACG motif-binding factor 6 | [ath04075](https://www.genome.jp/pathway/ath04075+AT5G06960)  Plant hormone signal transduction |
|  | AT5G06960 | transcription factor TGA \| (RefSeq) OBF5; OCS-element binding factor 5 | [ath04075](https://www.genome.jp/pathway/ath04075+AT5G06960)  Plant hormone signal transduction |
|  | AT3G02000 | ROXY1; Thioredoxin superfamily protein |  |
|  | AT1G32230 | RCD1; WWE protein-protein interaction domain protein family |  |
|  | AT5G14070 | ROXY2; Thioredoxin superfamily protein |  |
|  | AT2G47880 | Glutaredoxin family protein |  |
|  | AT2G25650 | DNA-binding storekeeper protein-related transcriptional regulator |  |
|  | AT4G33040 | Thioredoxin superfamily protein |  |
|  | AT5G06780 | EML2; Emsy N Terminus (ENT)/ plant Tudor-like domains-containing protein |  |
|  | AT2G30540 | Thioredoxin superfamily protein |  |

| **Supplementary Table S5**: TF interactions with other TFs or proteins were obtained from CORNET [34] based on IntACt, TAIR and AtPID databases. The metabolic pathways of each interacted protein were obtained from KEGG Pathway database [50]. | | | |
| --- | --- | --- | --- |
| TF name | Interacted protein 2 | Protein name | Metabolic pathway |
| bZIP45/TGA6 | AT4G19660 | regulatory protein NPR1 \| (RefSeq) NPR4; NPR1-like protein 4 |  |
|  | AT1G64280 | regulatory protein NPR1 \| (RefSeq) NPR1; regulatory protein (NPR1) |  |
|  | AT1G66410 | calmodulin \| (RefSeq) CAM4; calmodulin 4 | \| [ath04016](https://www.genome.jp/pathway/ath04016+AT1G66410) \| MAPK signaling pathway - plant \| \| --- \| --- \|  \| [ath04070](https://www.genome.jp/pathway/ath04070+AT1G66410) \| Phosphatidylinositol signaling system \| \| --- \| --- \|  \| [ath04626](https://www.genome.jp/pathway/ath04626+AT1G66410) \| Plant-pathogen interaction \| \| --- \| --- \| |
|  | AT1G28480 | GRX480; Thioredoxin superfamily protein |  |
|  | AT5G45110 | regulatory protein NPR1 \| (RefSeq) NPR3; NPR1-like protein 3 |  |
|  | AT5G06960 | transcription factor TGA \| (RefSeq) OBF5; OCS-element binding factor 5 | [ath04075](https://www.genome.jp/pathway/ath04075+AT5G06960)  Plant hormone signal transduction |
|  | AT5G06950 | transcription factor TGA \| (RefSeq) AHBP-1B; bZIP transcription factor family protein | [ath04075](https://www.genome.jp/pathway/ath04075+AT5G06960)  Plant hormone signal transduction |
|  | AT3G02000 | ROXY1; Thioredoxin superfamily protein |  |
|  | AT1G02450 | NIMIN1; NIM1-interacting 1 |  |
| GATA 11 | AT1G08000 | GATA10; GATA transcription factor 10 |  |
| HSF B-2b | AT1G45474 | light-harvesting complex I chlorophyll a/b binding protein 5 \| (RefSeq) Lhca5; photosystem I light harvesting complex protein 5 | \| [ath00196](https://www.genome.jp/pathway/ath00196+AT1G45474) \| Photosynthesis - antenna proteins \| \| --- \| --- \|  \| [ath01100](https://www.genome.jp/pathway/ath01100+AT1G45474) \| Metabolic pathways \| \| --- \| --- \| |
|  | AT5G43560 | TRAF-like superfamily protein |  |
|  | AT1G06680 | photosystem II oxygen-evolving enhancer protein 2 \| (RefSeq) PSBP-1; photosystem II subunit P-1 | \| [ath00195](https://www.genome.jp/pathway/ath00195+AT1G06680) \| Photosynthesis \| \| --- \| --- \|  \| [ath01100](https://www.genome.jp/pathway/ath01100+AT1G06680) \| Metabolic pathways \| \| --- \| --- \| |
| bHLH112 | AT4G02590 | UNE12; basic helix-loop-helix (bHLH) DNA-binding superfamily protein |  |
| NF-Y B-3 | AT2G34720 | nuclear transcription factor Y, alpha \| (RefSeq) NF-YA4; nuclear factor Y, subunit A4 |  |
