# Supplementary material for: Cold-responsive transcription factors in Arabidopsis and rice: A regulatory network analysis using array data and gene co-expression network
Source: PLoS One. 2023 Jun 8;18(6):e0286324. doi: 10.1371/journal.pone.0286324 (PMC10249815; doi:10.1371/journal.pone.0286324)
Supplement: S6 Table — (DOCX) [file pone.0286324.s006.docx]

| **Supplementary Table S6**: Predicted microRNA-TF in Arabidopsis- hybrid position, graph and mfe obtained using psRNATarget [37] and RNAhybrid [38]. | | | |
| --- | --- | --- | --- |
| TF name | *miRNA name* | Position | Mfe |
| ANT | *ath-miR5020c* | Position: 240  target 5' U AUG G G C 3'  UCUCAC UCU U AUGCCA  AGAGUG AGA G UACGGU  miRNA 3' C GA A G 5' | 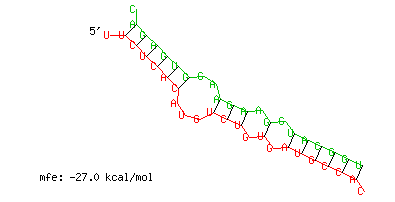 |
| ERF 4 | *ath-miR5646* | Position: 237  target 5' U UUU U 3'  UUCCCAAC UCUCGAGC  GAGGGUUG GGAGCUUG  miRNA 3' G CAC 5' | 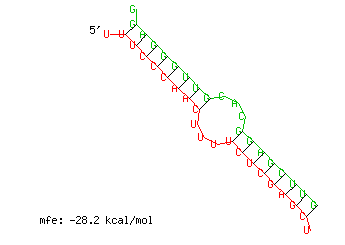 |
| ERF 5 | *ath-miR414* | Position: 696  target 5' A A C 3'  GACGAUGAUGA GAA GUGA  CUGCUACUACU CUU UACU  miRNA 3' A A C 5' | 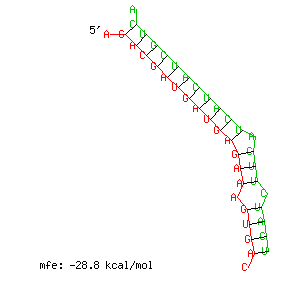 |
| ERF13 | *ath-miR391-5p* | Position: 191  target 5' C C G G 3'  CGUUA CUCUCC GCGGA  GCGAU GAGAGG CGCUU  miRNA 3' ACC A A 5' | 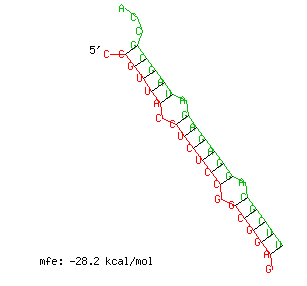 |

| **Supplementary Table S6**: Predicted microRNA-TF in Arabidopsis- hybrid position, graph and mfe obtained using psRNATarget [37] and RNAhybrid [38]. | | | |
| --- | --- | --- | --- |
| TF name | *miRNA name* | Position | Mfe |
| ERF38 | *ath-miR414* | Position: 93  target 5' A A 3'  GAUGAUGAUGAUGA GA  CUGCUACUACUACU CU  miRNA 3' A U ACU 5' | 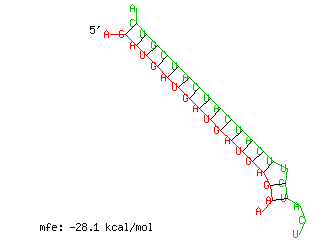 |
| ERF73 | *ath-miR8168* | Position: 477  target 5' G G A A 3'  GCG UGGUA GCUCAGCGCCU  CGU AUCGU UGAGUCGUGGA  miRNA 3' G G 5' | 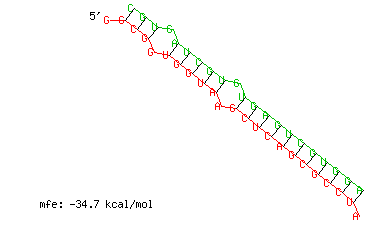 |
| ERF74-RAP2-12 | ath-miR396a-5p,b | Position: 458  target 5' C C A G 3'  GUUCAAGA AGCUG GGAA  CAAGUUCU UCGAC CCUU  miRNA 3' GU U A 5' | 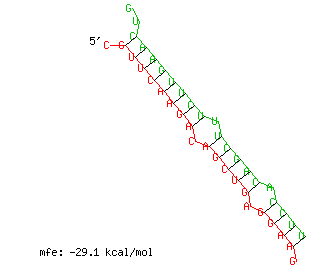 |
| ERF98 | ath-miR3932a,b | Position: 289  target 5' U C CG C 3'  CUUCGU GUCGUCAU GGUU  GAAGCA CAGUAGUG UCAA  miRNA 3' A UU 5' | 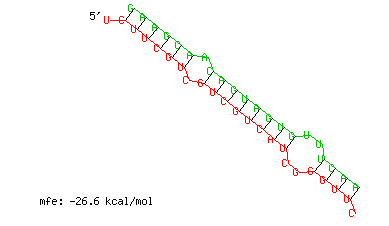 |

| **Supplementary Table S6**: Predicted microRNA-TF in Arabidopsis- hybrid position, graph and mfe obtained using psRNATarget [37] and RNAhybrid [38]. | | | |
| --- | --- | --- | --- |
| TF name | *miRNA name* | Position | Mfe |
| ERF113 | ath-miR5021 | Position: 518  target 5' C A C 3'  UUCUUC UCUUCUUCUU  AAGAAG AGAAGAAGAG  miRNA 3' AA A U 5' | 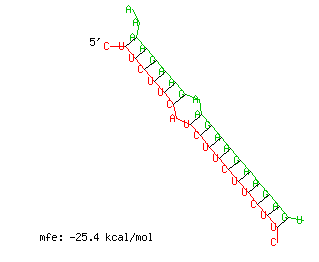 |
| MYB57 | ath-miR5654-3p | Position: 310  target 5' A G G 3'  AGAUU CGAAGCAUCUUCCG  UUUAG GUUUCGUAGAAGGU  miRNA 3' UUA G 5' | 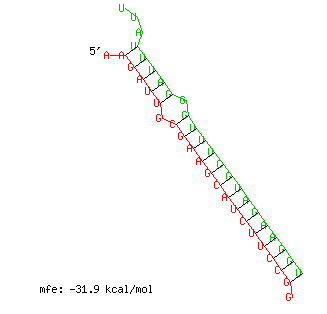 |
| MYB59 | ath-miR858a,b | Position: 215  target 5' C GGA U 3'  GG GAACAGAUAAUGAGA  CC CUUGUCUGUUGCUUU  miRNA 3' UU AG 5'  Position: 214  target 5' C A A 3'  CGGGG GAACAGAUAAUGAG  GUUCC CUUGUCUGUUGCUU  miRNA 3' AG 5' | 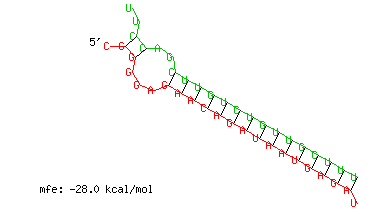 |

| **Supplementary Table S6**: Predicted microRNA-TF in Arabidopsis- hybrid position, graph and mfe obtained using psRNATarget [37] and RNAhybrid [38]. | | | |
| --- | --- | --- | --- |
| TF name | *miRNA name* | Position | Mfe |
| bHLH16/ UNE10b | *ath-miR838* | Position: 586  target 5' G C 3'  AAGAAG AGAAGAAAG  UUCUUC UCUUCUUUU  miRNA 3' ACACG A 5' | 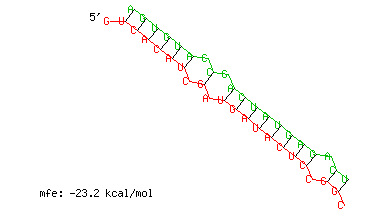 |
| bHLH35 | ath-miR1886.1 | Position: 440  target 5' G U U 3'  UUC UC CACUUCUCUCA  AAG AG GUGAAGAGAGU  miRNA 3' CUA U A 5' | 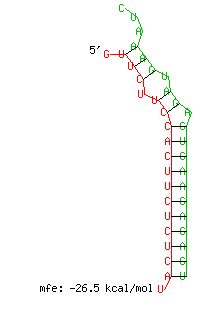 |
| bHLH 59/ UNE12 | ath-miR472-3p | Position: 189  target 5' C CU G 3'  AUGGGUGG UAGGAGGAA  UACCCGCC AUCCUUUUU  miRNA 3' CCA UC 5' | 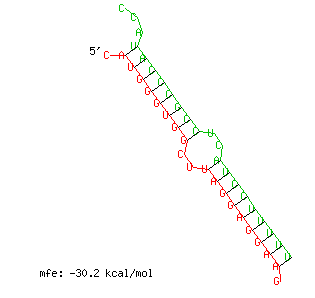 |
| bHLH79 | *ath-miR779.2* | Position: 595  target 5' C AUCAAUUAUA U 3'  UCGAUGAGAUU UUCAGUCA  AGUUGCUUUAA AGGUUAGU  miRNA 3' UC 5' | 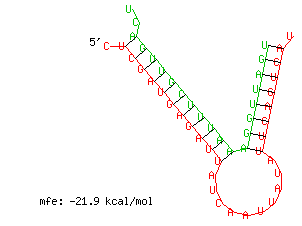 |

| **Supplementary Table S6**: Predicted microRNA-TF in Arabidopsis- hybrid position, graph and mfe obtained using psRNATarget [37] and RNAhybrid [38]. | | | |
| --- | --- | --- | --- |
| TF name | *miRNA name* | Position | Mfe |
| BHLH116/ICE1 | *ath-miR156a-3p* | osition: 1247  target 5' G A A 3'  GG AGGAAGAGCAGUGA  CU UCUUUCUCGUCACU  miRNA 3' AGA G CG 5' | 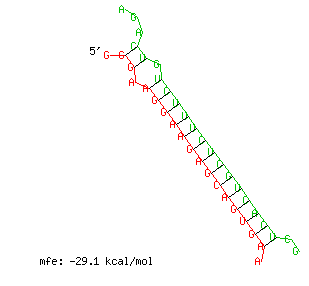 |
| bHLH102/BIM2 | *ath-miR867* | Position: 752  target 5' A CA U AAG A 3'  UG GGCA UGAUCUGUCGC C  AC UUGU AUUAGAUAGUG G  miRNA 3' G GA 5' | 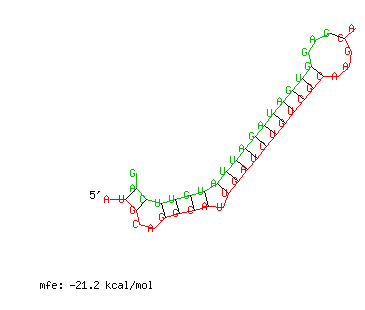 |
| bHLH105/ ILR3 | ath-miR3440b-3p | Position: 79  target 5' G U G 3'  GUUUCUCUUGGCC GUUCA  CGAAGGGAACUGG UAGGU  miRNA 3' UG U 5' | 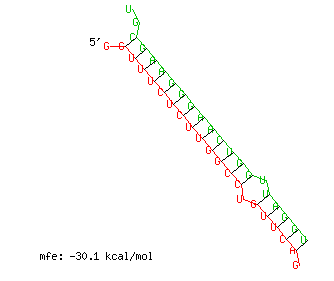 |
| bHLH128 | ath-miR395a,b,c,d,e,f | Position: 771  target 5' U CU A 3'  AGU UCCUUCAGACACUUCA  UCA AGGGGGUUUGUGAAGU  miRNA 3' C C 5' | 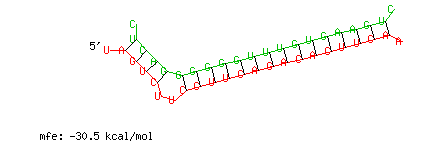 |

| **Supplementary Table S6**: Predicted microRNA-TF in Arabidopsis- hybrid position, graph and mfe obtained using psRNATarget [37] and RNAhybrid [38]. | | | | |
| --- | --- | --- | --- | --- |
| TF name | *miRNA name* | Position | Mfe | |
| bHLH129 | ath-miR779.1 | Position: 145  target 5' C C U 3'  AGCAGCAGCAU AGCAGAA  UCGUCGUUGUA UCGUCUU  miRNA 3' UAC 5' | | 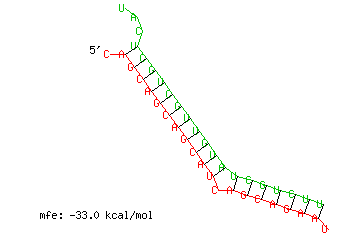 |
| bHLH137 | ath-miR5023 | Position: 110  target 5' A A 3'  UCUC UAUCCACUACCAA  GGGG AUAGGUGAUGGUU  miRNA 3' CG A A 5' | | 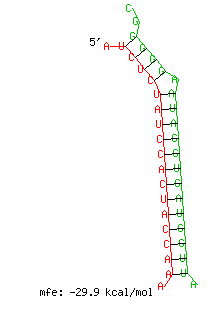 |
| bHLH148 | ath-miR870-3p | Position: 145  target 5' C ACAA C 3'  GAUCGGAGAAGCA CGGAU  CUAGCUUCUUUGU GUUUA  miRNA 3' G AU 5' | | 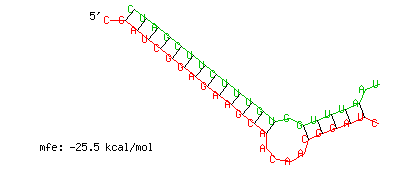 |
| NFYA-10 | ath-miR836 | Position: 496  target 5' A AAACU AAU G 3'  CCAU CAUCGAGGGAAGCA GA  GGUG GUAGUUUCCUUUGU CU  miRNA 3' C GUC 5' | | 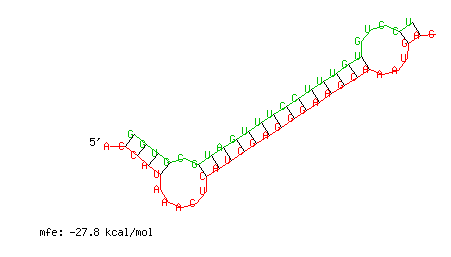 |

| **Supplementary Table S6**: Predicted microRNA-TF in Arabidopsis- hybrid position, graph and mfe obtained using psRNATarget [37] and RNAhybrid [38]. | | | |
| --- | --- | --- | --- |
| TF name | *miRNA name* | Position | Mfe |
| bZIP20/TGA2 | ath-miR3434-3p | Position: 316  target 5' A UG AAA C 3'  C GUGG UGGUGCUUUGG  G UACC ACUAUGAGACU  miRNA 3' A UG G 5' | 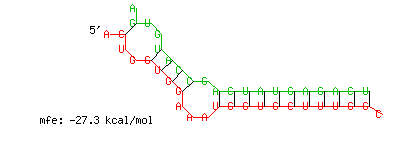 |
| bZIP45/TGA6 | *ath-miR859* | Position: 647  target 5' A G C A 3'  GAC UCGCAGCAGG AGA  CUG AGUGUUGUCU UCU  miRNA 3' AAA A C 5' | 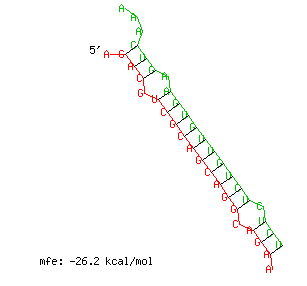 |
| bZIP 60 | ath-miR414 | osition: 383  target 5' A C AG G 3'  UGAUGAUGAUGA GAAGA GA  ACUGCUACUACU CUUCU CU  miRNA 3' A A 5' | 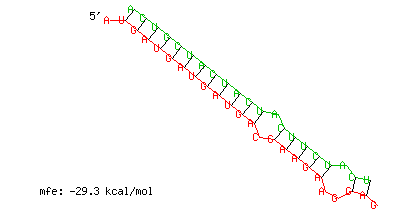 |

| **Supplementary Table S6**: Predicted microRNA-TF in Arabidopsis- hybrid position, graph and mfe obtained using psRNATarget [37] and RNAhybrid [38]. | | | | |
| --- | --- | --- | --- | --- |
| TF name | *miRNA name* | Position | | Mfe |
| GATA 11 | *ath-miR5020a* | osition: 589  target 5' C A GACCACCCGCACGG CU C 3'  GCAAG UC CACCU UCU UCCA  CGUUC AG GUGGA AGA AGGU  miRNA 3' A A 5' | | 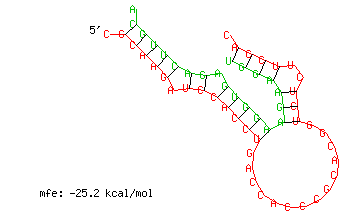 |
| GATA 22 | *ath-miR8172* | Position: 625  target 5' A UC GAAG A 3'  CUCC UUUGGA UGGUCCG  GAGG AGAUCU ACUAGGU  miRNA 3' UA U A 5' | | 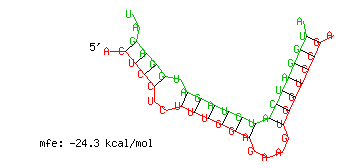 |
| GATA 23 | ath-miR5020b | Position: 26  target 5' G C A G 3'  UUCAUC UCUU CGUGUCA  GAGUGG AGAA GUACGGU  miRNA 3' A A A A 5' | 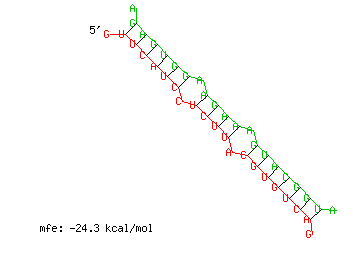 | |
| HSF A-3 | ath-miR156b-3p | Position: 798  target 5' A G 3'  ACAGGAGGGGAGGUG GU UGUCUUUCUCUCCAC CG  miRNA 3' UGAC U U 5' | 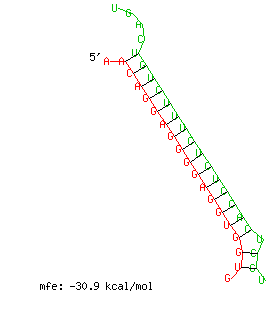 | |
| **Supplementary Table S6**: Predicted microRNA-TF in Arabidopsis- hybrid position, graph and mfe obtained using psRNATarget [37] and RNAhybrid [38]. | | | | |
| TF name | *miRNA name* | Position | Mfe | |
| HSF A-9 | ath-miR395b,c,f | Position: 312  target 5' C CAGAGAAUCUACUU A 3'  GAGUUCU CCCAAAUACUUCA  CUCAGGG GGGUUUGUGAAGU  miRNA 3' C 5' | 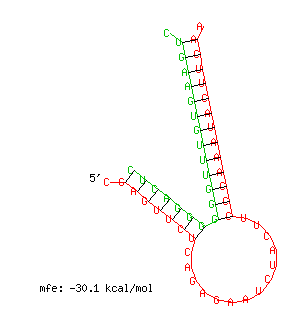 | |
| HSF B-2b | ath-miR834 | Position: 602  target 5' C G U 3'  UGC GCUGCUGCUGCUGCUA  AUG UGGCGAUGACGAUGGU  miRNA 3' A G 5' | 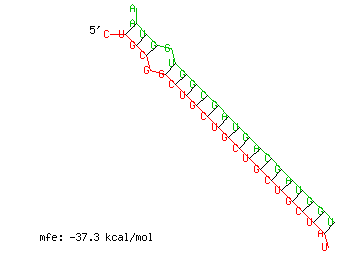 | |
| WRKY1/ZAP1 | ath-miR771 | Position: 978  target 5' A A U A 3'  AGG GCUACUAUAGA GUUCA  UCC CGAUGGUGUCU CGAGU  miRNA 3' AC C 5' | 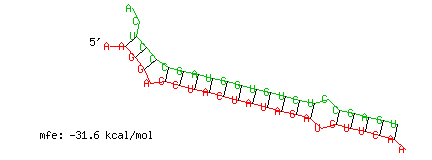 | |

| **Supplementary Table S6**: Predicted microRNA-TF in Arabidopsis- hybrid position, graph and mfe obtained using psRNATarget [37] and RNAhybrid [38]. | | | |
| --- | --- | --- | --- |
| TF name | *miRNA name* | Position | Mfe |
| GATA 11 | *ath-miR5020a* | Position: 589  target 5' C A GACCACCCGCACGG CU C 3'  GCAAG UC CACCU UCU UCCA  CGUUC AG GUGGA AGA AGGU  miRNA 3' A A 5' | 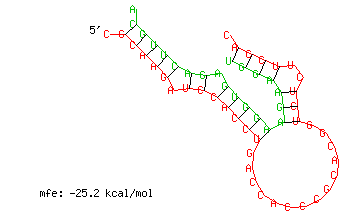 |
| GATA 22 | *ath-miR8172* | Position: 625  target 5' A UC GAAG A 3'  CUCC UUUGGA UGGUCCG  GAGG AGAUCU ACUAGGU  miRNA 3' UA U A 5' | 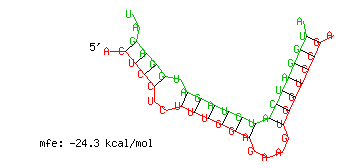 |
| GATA 23 | ath-miR5020b | Position: 26  target 5' G C A G 3'  UUCAUC UCUU CGUGUCA  GAGUGG AGAA GUACGGU  miRNA 3' A A A A 5' | 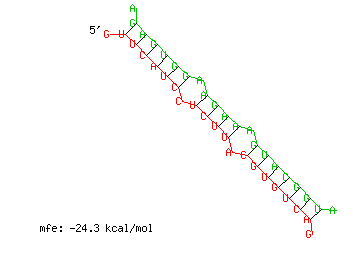 |
| HSF A-3 | ath-miR156b-3p | Position: 798  target 5' A G 3'  ACAGGAGGGGAGGUG GU UGUCUUUCUCUCCAC CG  miRNA 3' UGAC U U 5' | 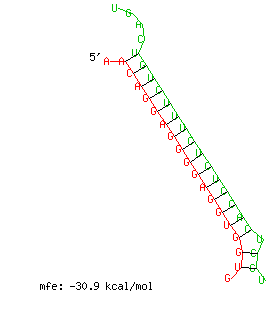 |
| **Supplementary Table S6**: Predicted microRNA-TF in Arabidopsis- hybrid position, graph and mfe obtained using psRNATarget [37] and RNAhybrid [38]. | | | |
| TF name | *miRNA name* | Position | Mfe |
| PLT2 | *ath-miR5658* | Position: 1287  target 5' G U G 3'  CAUC UCAUCAUCAUCA  GUAG AGUAGUAGUAGU  miRNA 3' AAA U A 5' | 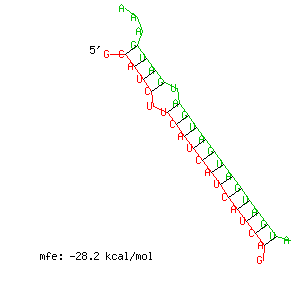 |
| ERF39 | ath-miR855 | Position: 435  target 5' C GAUC GAU U 3'  UCC UUCCU CUUUUGCU  AGG AAGGA GAAAACGA  miRNA 3' A AA AUC 5' | 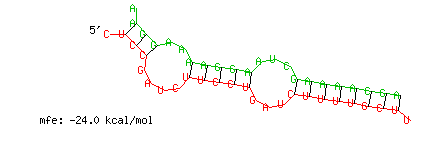 |
| ERF54 | *ath-miR5020a* | Position: 830  target 5' A C 3'  AAG CUCAUCUUCUUCU  UUC GAGUGGAAGAAGG  miRNA 3' ACG A U 5' | 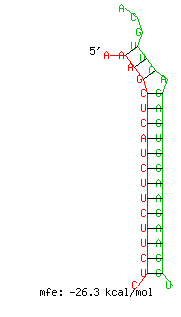 |
| MYB5 | *ath-miR171c-5p* | Position: 542  target 5' A A U 3'  GAAC GUAUCAAUGUCU  CUUG CGUGGUUAUAGA  miRNA 3' CUAA G 5' | 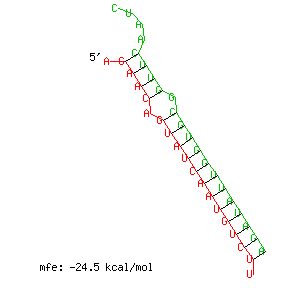 |

| **Supplementary Table S6**: Predicted microRNA-TF in Arabidopsis- hybrid position, graph and mfe obtained using psRNATarget [37] and RNAhybrid [38]. | | | |
| --- | --- | --- | --- |
| TF name | *miRNA name* | Position | Mfe |
| GATA 11 | *ath-miR5020a* | osition: 589  target 5' C A GACCACCCGCACGG CU C 3'  GCAAG UC CACCU UCU UCCA  CGUUC AG GUGGA AGA AGGU  miRNA 3' A A 5' | 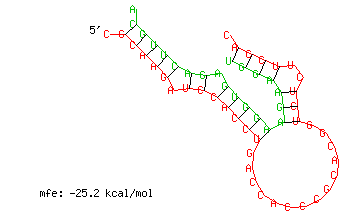 |
| GATA 22 | *ath-miR8172* | Position: 625  target 5' A UC GAAG A 3'  CUCC UUUGGA UGGUCCG  GAGG AGAUCU ACUAGGU  miRNA 3' UA U A 5' | 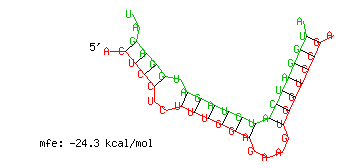 |
| GATA 23 | ath-miR5020b | Position: 26  target 5' G C A G 3'  UUCAUC UCUU CGUGUCA  GAGUGG AGAA GUACGGU  miRNA 3' A A A A 5' | 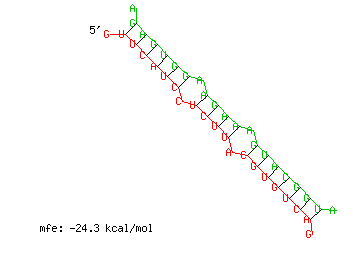 |
| HSF  A-3 | ath-miR156b-3p | Position: 798  target 5' A G 3'  ACAGGAGGGGAGGUG GU UGUCUUUCUCUCCAC CG  miRNA 3' UGAC U U 5' | 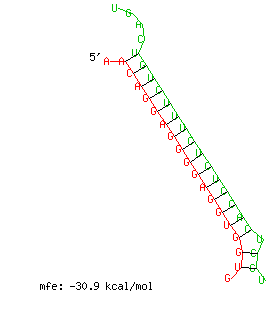 |

| **Supplementary Table S6**: Predicted microRNA-TF in Arabidopsis- hybrid position, graph and mfe obtained using psRNATarget [37] and RNAhybrid [38]. | | | |
| --- | --- | --- | --- |
| TF name | *miRNA name* | Position | Mfe |
| MYB37/RAX1 | *ath-miR858a,b* | Position: 293  target 5' C A C 3'  GGG CGAACAGACAACGA  UCC GCUUGUCUGUUGCU  miRNA 3' U A UU 5' | 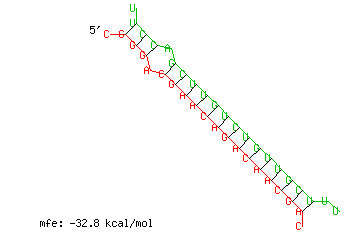 |
| MYB38/RAX2 | *ath-miR5658* | Position: 725  target 5' A G G 3'  U AUCAUCAUCAUCAUCAU  A UAGUAGUAGUAGUAGUA  miRNA 3' AA G 5' | 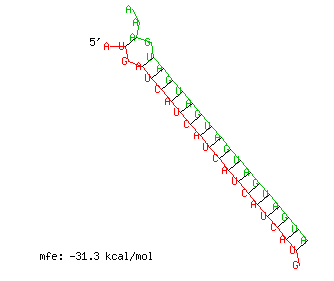 |
| MYB44 | *ath-miR5016* | Position: 20  target 5' G U UG G 3'  UCCA GGAGUCC AAGAA  AGGU CCUUAGG UUCUU  miRNA 3' AA U UG 5' | 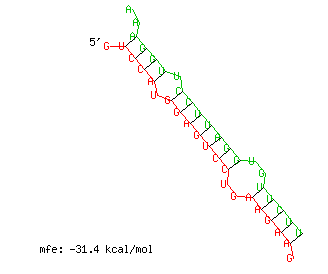 |

| **Supplementary Table S6**: Predicted microRNA-TF in Arabidopsis- hybrid position, graph and mfe obtained using psRNATarget [37] and RNAhybrid [38]. | | | |
| --- | --- | --- | --- |
| TF name | *miRNA name* | Position | Mfe |
| MYB84/RAX3 | ath-miR858a,b | Position: 293  target 5' C A U 3'  GGG CGAACAGACAACGA  UCC GCUUGUCUGUUGCU  miRNA 3' U A UU 5' | 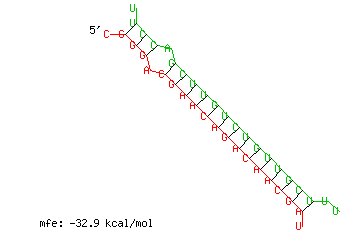 |
| bHLH112 | *ath-miR391-5p* | Position: 71  target 5' G A G U U 3'  G GCGUUAU UCUCCU CGGA  C CGCGAUA AGAGGA GCUU  miRNA 3' A G C 5' | 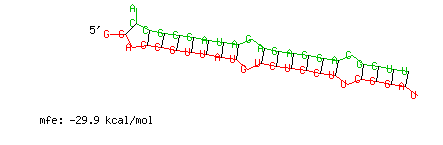 |
| bHLH113 | *ath-miR771* | Position: 2  target 5' U AGA A G 3'  GGG UACCGCAGAGG UCA  CCC AUGGUGUCUCC AGU  miRNA 3' ACU G G 5' | 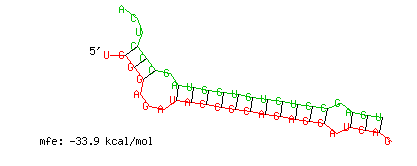 |
|  |  |  |  |

| **Supplementary Table S6**: Predicted microRNA-TF in Arabidopsis- hybrid position, graph and mfe obtained using psRNATarget [37] and RNAhybrid [38]. | | | |
| --- | --- | --- | --- |
| TF name | *miRNA name* | Position | Mfe |
| NF-Y B-3 | *ath-miR157c-3* | Position: 393  target 5' C AGCU GGAA G U 3'  GGUGG GGAAGU GUGGA GAGC  CCACU UCUUCA UAUCU CUCG  miRNA 3' G 5' | 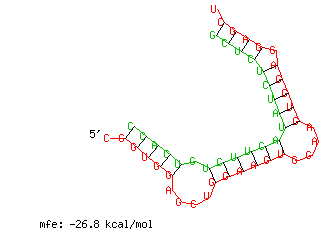 |
| NF-Y B-4 | *ath-miR5012* | Position: 294  target 5' U ACAAA AAUGAU A 3'  GAGCACA GGUAGC AGUGGGA  CUUGUGU UCAUCG UCAUUUU  miRNA 3' C 5' | 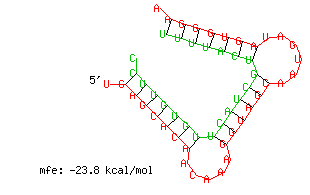 |
| NF-Y B-9 | *ath-miR854a,b,c,d,e* | Position: 546  target 5' A GG G 3'  CCUCCUCC GUCCUUAU  GGAGGAGG UAGGAGUA  miRNA 3' GA GA G 5' | 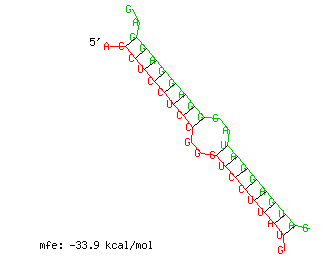 |
| NF-Y C-2 | *ath-miR414* | Position: 263  target 5' C A U 3'  UGAUGA GAUG UGAGGAUGA  ACUGCU CUAC ACUUCUACU  miRNA 3' A U 5' | 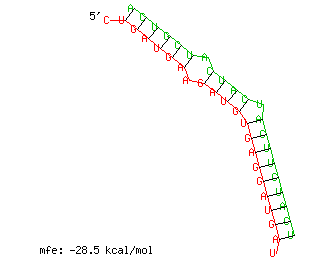 |

| **Supplementary Table S6**: Predicted microRNA-TF in Arabidopsis- hybrid position, graph and mfe obtained using psRNATarget [37] and RNAhybrid [38]. | | | |
| --- | --- | --- | --- |
| TF name | *miRNA name* | Position | Mfe |
| bZIP17 | *ath-miR834* | Position: 1803  target 5' A AUUC A A 3'  GCCAUC CUGC GCUACCA  UGGUGG GAUG CGAUGGU  miRNA 3' AA C A 5' | 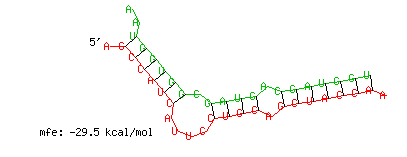 |
| TCP21 | *ath-miR5658* | Position: 582  target 5' A GCAACCAUUGUUUGU G 3'  CAUCAUCAUCA UCAUCA  GUAGUAGUAGU AGUAGU  miRNA 3' AAA A 5' | 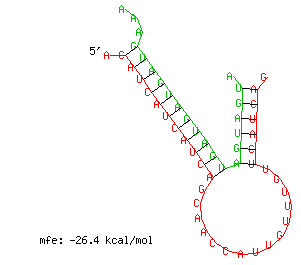 |

| **Supplementary Table S6**: Predicted microRNA-TF in Arabidopsis- hybrid position, graph and mfe obtained using psRNATarget [37] and RNAhybrid [38]. | | | |
| --- | --- | --- | --- |
| TF name | *miRNA name* | Position | Mfe |
| ANT | osa-miR6255 | *Position: 87*  *target 5' G CAACAU CUG U 3'*  *GCUCG CUGCCCAU UCUCA*  *UGAGU GACGGGUA AGGGU*  *miRNA 3' U AAA 5'* | 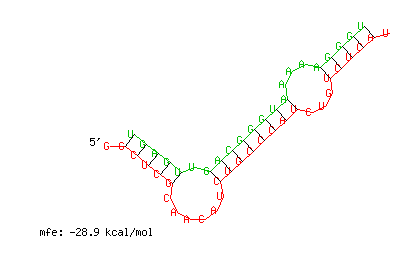 |
| ERF 4 | osa-miR531a,c | *Position: 770*  *target 5' C GGAG G AGG G 3'*  *GGCGGC GCG GGCU CGGCGAG*  *CCGCCG UGC UCGG GCCGCUC*  *miRNA 3' UA G G 5'* | 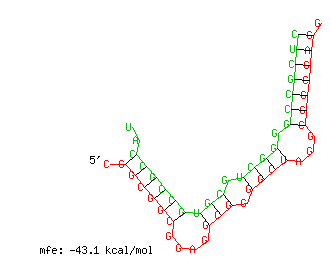 |
| ERF 5 | *osa-miR437* | *Position: 502*  *target 5' A C G 3'*  *CAAAC UCUCUAAUUUU*  *GUUUG AGAGAUUGAAA*  *miRNA 3' UUCA A 5'* | 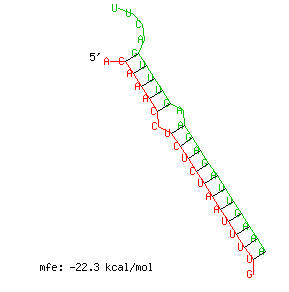 |
| ERF13 | osa-miR2094-3p | *Position: 363*  *target 5' G UG C GAAACGGC G 3'*  *G G UGGA UGCCACGGCUCUG*  *C C ACCU ACGGUGUCGAGAC*  *miRNA 3' G UG 5'* | 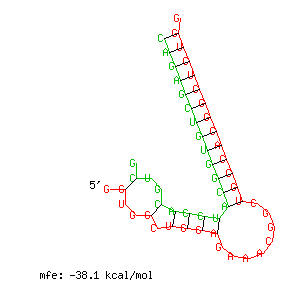 |

| **Supplementary Table S6**: Predicted microRNA-TF in Arabidopsis- hybrid position, graph and mfe obtained using psRNATarget [37] and RNAhybrid [38]. | | | |
| --- | --- | --- | --- |
| TF name | *miRNA name* | Position | Mfe |
| ERF38 | osa-miR439a-b-c-d-e-f-g-h-i | *Position: 514*  *target 5' G U C A 3'*  *UCGAGC GCCGCGG UCGACG*  *AGCUUG UGGCGCC AGCUGU*  *miRNA 3' U A 5'* | 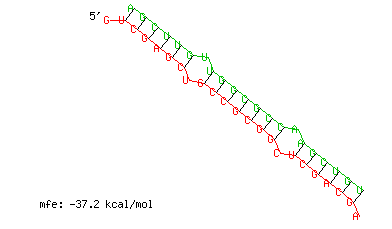 |
| ERF73 | *osa-miR5075* | *Position: 299*  *target 5' A CG A 3'*  *GCGG GCGGCGGCGGCGGAGGG*  *CGCC UGCCGCCGCUGCCUCUU*  *miRNA 3' 5'* | 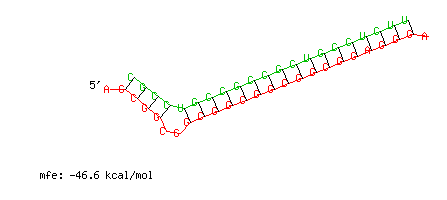 |
| ERF74-RAP2-12 | *osa-*  *miR2102-3p* | *Position: 75*  *target 5' U C G 3'*  *GUCGCCGGAGCCG CGCCGUG*  *CGGUGGCCUUGGC GUGGUAC*  *miRNA 3' G C 5'* | 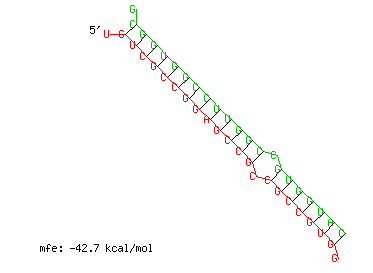 |
| ERF98 | *osa-miR5075* | *Position: 236*  *target 5' C A U U 3'*  *CG GGCGGCGGCGGCGG GG*  *GC CUGCCGCCGCUGCC CU*  *miRNA 3' C U U 5'* | 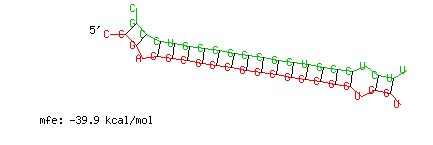 |

| **Supplementary Table S6**: Predicted microRNA-TF in Arabidopsis- hybrid position, graph and mfe obtained using psRNATarget [37] and RNAhybrid [38]. | | | |
| --- | --- | --- | --- |
| TF name | *miRNA name* | Position | Mfe |
| ERF113 | *osa-miR5075* | *Position: 279*  *target 5' G GCUUC G 3'*  *GCGGA UGGUGGCGGCGGAGAG*  *CGCCU GCCGCCGCUGCCUCUU*  *miRNA 3' 5'* | 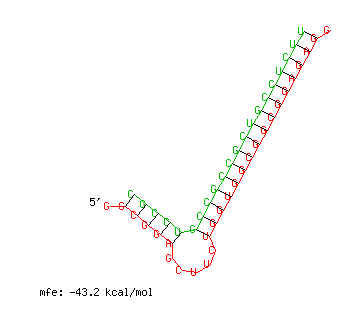 |
| DREB 1A | *osa-miR5075* | *Position: 304*  *target 5' G G 3'*  *GCGG CGGCGGCGGCGGGGGA*  *CGCC GCCGCCGCUGCCUCUU*  *miRNA 3' U 5'* | 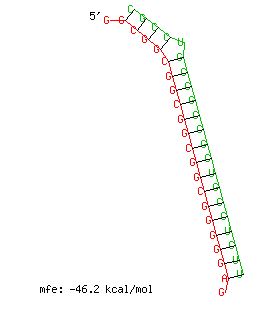 |
| DREB 1B | *osa-miR5075* | *Position: 466*  *target 5' C U 3'*  *CGG UGGCGGCGGCGGAGG*  *GCC GCCGCCGCUGCCUCU*  *miRNA 3' C U U 5'* | 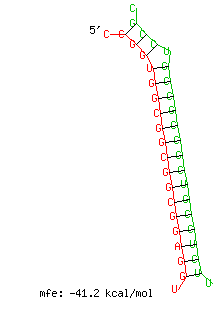 |
| MYB57 | *osa-miR5832* | ***Position: 446***  ***target 5' A A G G 3'***  ***GCAGCAGC GCUC GCCAA***  ***UGUCGUUG CGAG CGGUU***  ***miRNA 3' AC G G 5'*** | 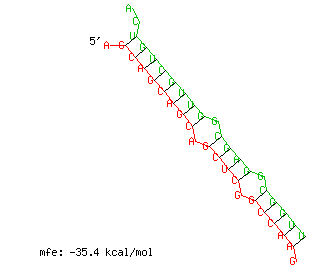 |

| **Supplementary Table S6**: Predicted microRNA-TF in Arabidopsis- hybrid position, graph and mfe obtained using psRNATarget [37] and RNAhybrid [38]. | | | |
| --- | --- | --- | --- |
| TF name | *miRNA name* | Position | Mfe |
| MYB59 | *osa-miR5833* | *Position: 140*  *target 5' G C G 3'*  *GAUGAG CCCGAGGAGGA*  *CUACUC GGGCUCCUCCU*  *miRNA 3' CGGG 5'* | 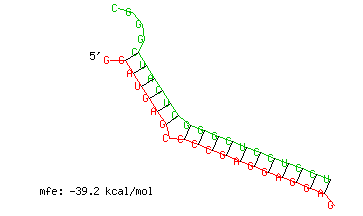 |
| bHLH16/ UNE10b | *osa-miR5075* | *Position: 25*  *target 5' G CG G 3'*  *GCGG GCGGCGGCGGCGGGGAG*  *CGCC UGCCGCCGCUGCCUCUU*  *miRNA 3' 5'* | 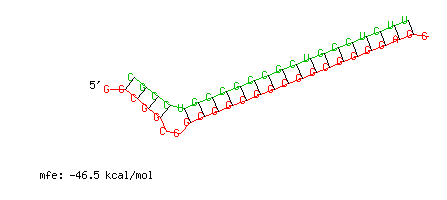 |
| bHLH35 | *osa-miR414* | *Position: 317*  *target 5' U CAGGGUGC C C 3'*  *GGGCGA UGGUGGUGAG GUGA*  *CCUGCU ACUACUACUC UACU*  *miRNA 3' C 5'* | 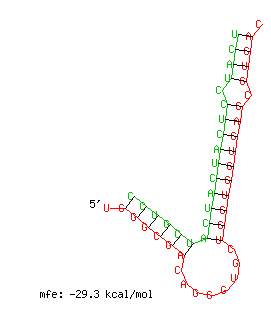 |
| bHLH 59/ UNE12 | *osa-miR5075* | *Position: 38*  *target 5' U A C 3'*  *CGG CGGCGGCGGCG GGA*  *GCC GCCGCCGCUGC UCU*  *miRNA 3' C U C U 5'* | 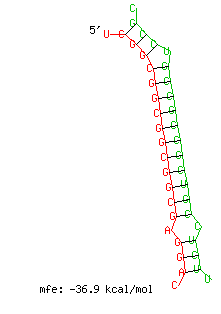 |

| **Supplementary Table S6**: Predicted microRNA-TF in Arabidopsis- hybrid position, graph and mfe obtained using psRNATarget [37] and RNAhybrid [38]. | | | |
| --- | --- | --- | --- |
| TF name | *miRNA name* | Position | Mfe |
| bHLH79 | *osa-miR5075* | *Position: 217*  *target 5' A CA A C 3'*  *GCGG GC GCGGCGGCGGAGG*  *CGCC UG CGCCGCUGCCUCU*  *miRNA 3' C U 5'* | 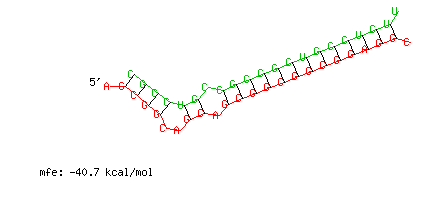 |
| bHLH102/BIM2 | *osa-miR5075* | *Position: 448*  *target 5' G C 3'*  *GGGCGGCGGCGGCGGAGA*  *CCUGCCGCCGCUGCCUCU*  *miRNA 3' CG U 5'* | 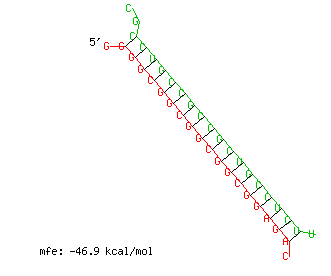 |
| bHLH105/ ILR3 | *osa-miR5075* | *Position: 15*  *target 5' G C 3'*  *GCGGACGGCGGCGGCGGAGG*  *CGCCUGCCGCCGCUGCCUCU*  *miRNA 3' U 5'* | 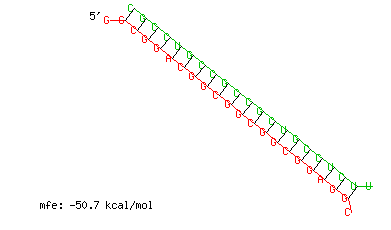 |
| BHLH116/ICE1 | *osa-miR1848* | *Position: 337*  *target 5' C UGUA GA A 3'*  *UGCACGCGC CG GGCGGGG*  *ACGUGCGCG GC CCGCUCC*  *miRNA 3' C GG 5'* | 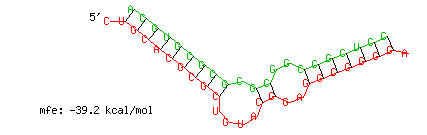 |
| bHLH128 | *osa-miR408-3p* | *Position: 692*  *target 5' U A U 3'*  *GCU GGAGGA GCAGUGCAG*  *CGG CCUUCU CGUCACGUC*  *miRNA 3' UC C 5'* | 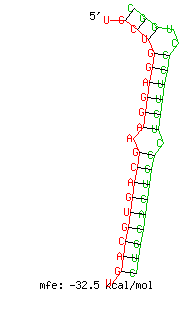 |

| **Supplementary Table S6**: Predicted microRNA-TF in Arabidopsis- hybrid position, graph and mfe obtained using psRNATarget [37] and RNAhybrid [38]. | | | |
| --- | --- | --- | --- |
| TF name | *miRNA name* | Position | Mfe |
| bHLH129 | *osa-miR5493* | *Position: 111*  *target 5' G A CCAGUACUACG G 3'*  *CACG CGCGGCCGA GCUCGGC*  *GUGC GCGCUGGCU CGGGCCG*  *miRNA 3' A 5'* | 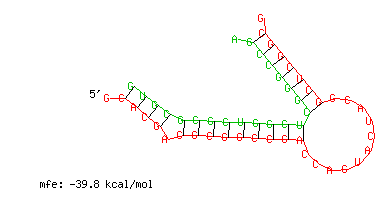 |
| bHLH137 | *osa-miR5515* | *Position: 43*  *target 5' A UG C C 3'*  *C CUG UGGAGCAGCCAUCG*  *G GAC AUCUUGUUGGUAGC*  *miRNA 3' UG C 5'* | 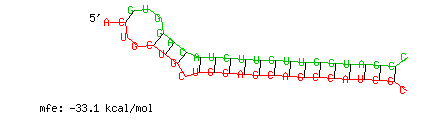 |
| bHLH148 | *osa-miR5075* | *Position: 274*  *target 5' G G 3'*  *GCGG CGGCGGCGGCGGGGGG*  *CGCC GCCGCCGCUGCCUCUU*  *miRNA 3' U 5'* | 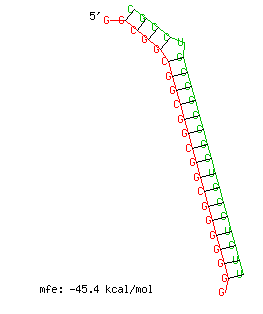 |
| NFYA-4 | *osa-miR5075* | *Position: 170*  *target 5' G GACGACGCGGCAGA C 3'*  *GGAUGGCGGCGGC GGAGAG*  *CCUGCCGCCGCUG CCUCUU*  *miRNA 3' CG 5'* | 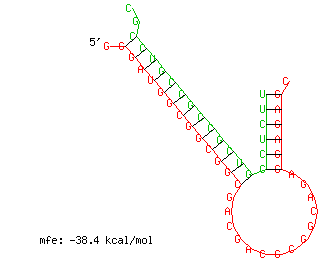 |

| **Supplementary Table S6**: Predicted microRNA-TF in Arabidopsis- hybrid position, graph and mfe obtained using psRNATarget [37] and RNAhybrid [38]. | | | |
| --- | --- | --- | --- |
| TF name | *miRNA name* | Position | Mfe |
| NFYA-10 | *osa-miR2873a* | *Position: 543*  *target 5' A AAAG C U 3'*  *GCUAGA UAAGUCU AACUU*  *UGGUUU AUUCAGG UUGAA*  *miRNA 3' CAA AA U 5'* | 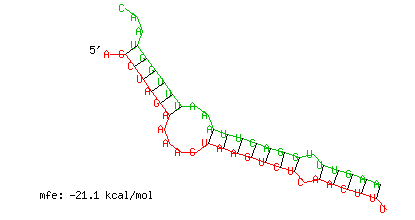 |
| bZIP20/TGA2 | *osa-miR5075* | *Position: 42*  *target 5' G C 3'*  *GCGG CGGCGGCGGCGGA*  *CGCC GCCGCCGCUGCCU*  *miRNA 3' U CUU 5'* | 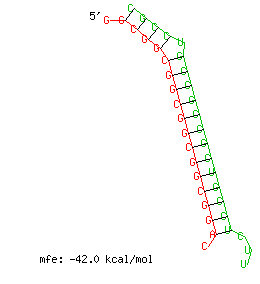 |
| bZIP45/TGA6 | *osa-miR171i-5p*  *osa-miR172d-5p* | *Position: 650*  *target 5' C A C 3'*  *AUUG GGCAC CCAAUAUCU*  *UAAC CCGUG GGUUAUGGA*  *miRNA 3' C U C 5'* | 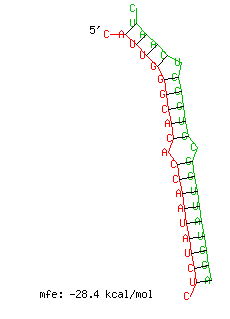  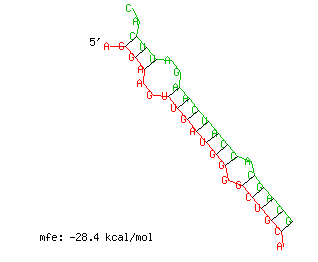 |

| **Supplementary Table S6**: Predicted microRNA-TF in Arabidopsis- hybrid position, graph and mfe obtained using psRNATarget [37] and RNAhybrid [38]. | | | |
| --- | --- | --- | --- |
| TF name | *miRNA name* | Position | Mfe |
| bZIP 60 | *osa-miR5795* | *Position: 98*  *target 5' C A U A 3'*  *GCUGG GGA UUCGAUCUCGACG*  *CGGCC CUU GAGCUGGAGCUGU*  *miRNA 3' A 5'* | 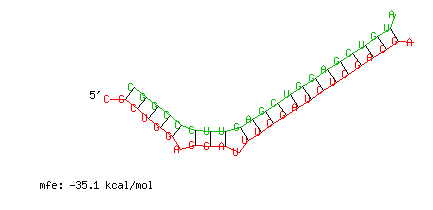 |
| GATA 11 | *osa-miR2925* | *Position: 636*  *target 5' G U G U A 3'*  *GCGA G CCGC GCUGCCA*  *UGCU C GGCG CGGCGGU*  *miRNA 3' U G C 5'* | 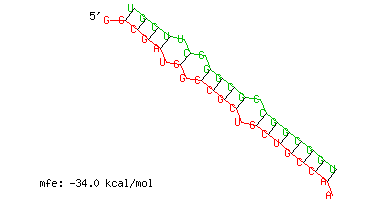 |
| GATA 22 | *osa-miR528-3p* | *Position: 529*  *target 5' A C C 3'*  *GGAGGAGGC GGCGCAGG*  *CCUUCUCCG UCGUGUCC*  *miRNA 3' UUA U 5'* | 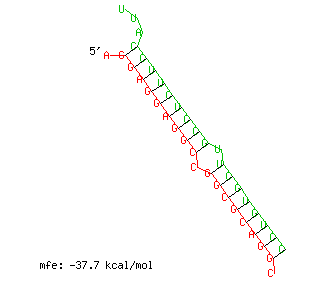 |
| GATA 23 | *osa-miR168b* | *Position: 80*  *target 5' A UU G UGAACC A 3'*  *UC GGGCUG ACC AGCC*  *AG CUCGAC UGG UCGG*  *miRNA 3' A GG G U A 5'* | 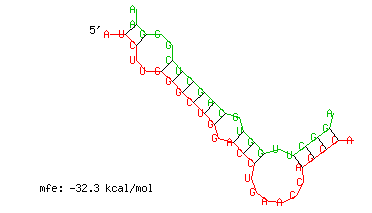 |

| **Supplementary Table S6**: Predicted microRNA-TF in Arabidopsis- hybrid position, graph and mfe obtained using psRNATarget [37] and RNAhybrid [38]. | | | |
| --- | --- | --- | --- |
| TF name | *miRNA name* | Position | Mfe |
| HSF A-3 | *osa-miR5075* | *Position: 90*  *target 5' C A 3'*  *GACGGCGGCGGCG*  *CUGCCGCCGCUGC*  *miRNA 3' CGC CUCUU 5'* | 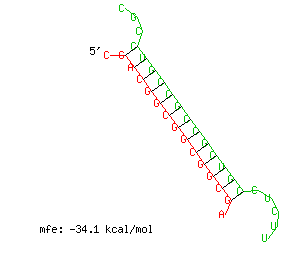 |
| HSF A-9 | *osa-miR5075* | *Position: 61*  *target 5' G C G 3'*  *GCGG CGGCGGCGGCGG GAG*  *CGCC GCCGCCGCUGCC CUU*  *miRNA 3' U U 5'* | 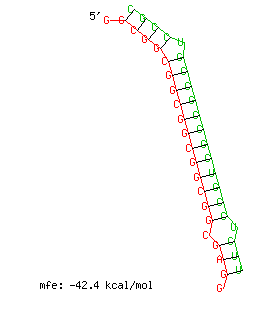 |
| HSF B-2b | *osa-miR5075* | *Position: 61*  *target 5' G C G 3'*  *GCGG CGGCGGCGGCGG GAG*  *CGCC GCCGCCGCUGCC CUU*  *miRNA 3' U U 5'* | 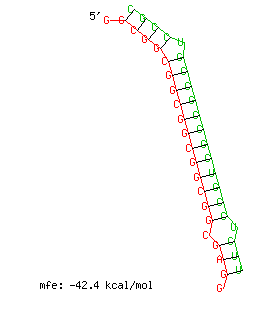 |
| HSF B4 | *osa-miR5075* | *Position: 61*  *target 5' G CG GC G 3'*  *GCGG GCGGC GGCGGCGGGGAA*  *CGCC UGCCG CCGCUGCCUCUU*  *miRNA 3' 5'* | 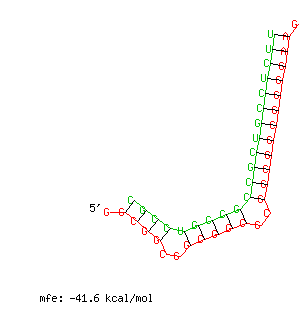 |

| **Supplementary Table S6**: Predicted microRNA-TF in Arabidopsis- hybrid position, graph and mfe obtained using psRNATarget [37] and RNAhybrid [38]. | | | |
| --- | --- | --- | --- |
| TF name | *miRNA name* | Position | Mfe |
| WRKY1/ZAP1 | *osa-miR5493* | *Position: 1319*  *target 5' C GCG G 3'*  *CGCGCGGCCGGGC CCGGC*  *GCGCGCUGGCUCG GGCCG*  *miRNA 3' GU A 5'* | 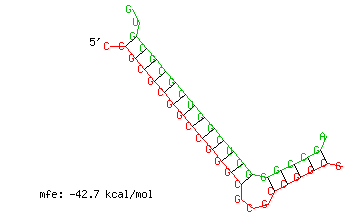 |
| PLT2 | *osa-miR2094-5p* | Position: 506  target 5' U AAU A C 3'  CAC GGGG CUAGCAGCCA  GUG CCUC GAUCGUCGGU  miRNA 3' GGU G 5' | 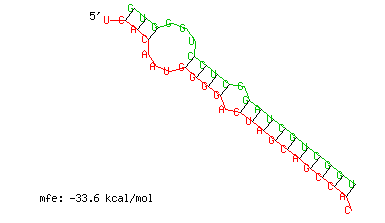 |
| ERF39 | *osa-miR5075* | Position: 95  target 5' A CGU C 3'  GGA CGGCGGCGGCGGGGAG  CCU GCCGCCGCUGCCUCUU  miRNA 3' CG 5' | 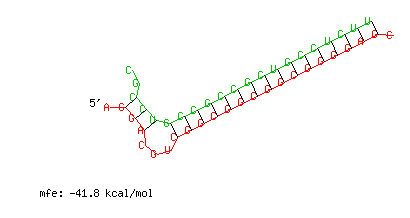 |
| ERF54 | *osa-miR6246* | *Position: 540*  *target 5' G CG U UC C 3'*  *UCCU CCGGCGG G GUCCCCGA*  *AGGA GGCCGUC C UAGGGGUU*  *miRNA 3' A UU 5'* | 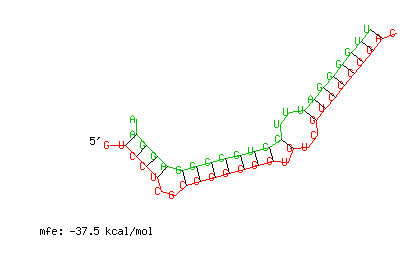 |

| **Supplementary Table S6**: Predicted microRNA-TF in Arabidopsis- hybrid position, graph and mfe obtained using psRNATarget [37] and RNAhybrid [38]. | | | |
| --- | --- | --- | --- |
| TF name | *miRNA name* | Position | Mfe |
| MYB5 | *osa-miR159c* | Position: 808  target 5' A U 3'  UGGAGCUCCCUUUAAUCCAAU  ACCUCGAGGGAAGUUAGGUUA  miRNA 3' 5' | 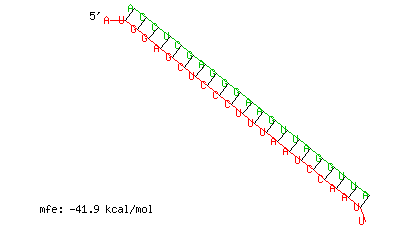 |
| MYB37/RAX1 | *osa-miR2104* | Position: 390  target 5' G CG C 3'  GCGC CGC CCCCUCGCCGC  UGCG GCG GGGGAGCGGCG  miRNA 3' G A UA 5' | 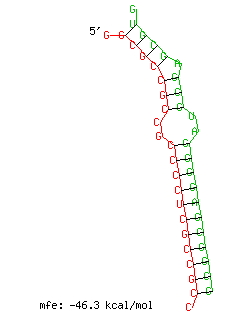 |
| MYB38/RAX2 | *osa-miR2925* | *Position: 543*  *target 5' C GG C C 3'*  *GCG GCCCGU GCCGCCG*  *UGC CGGGCG CGGCGGU*  *miRNA 3' UU C 5'* |  |
| MYB44 | *osa-miR2927* | *Position: 634*  *target 5' U U GUCG GGA A 3'*  *CGUGG C CCGUCGACG ACGACA*  *GUACC G GGUAGCUGC UGCUGU*  *miRNA 3' C A 5'* |  |

| **Supplementary Table S6**: Predicted microRNA-TF in Arabidopsis- hybrid position, graph and mfe obtained using psRNATarget [37] and RNAhybrid [38]. | | | |
| --- | --- | --- | --- |
| TF name | *miRNA name* | Position | Mfe |
| MYB84/RAX3 | *osa-miR1846a-5p,b,c* | *Position: 593*  *target 5' C C U GA C 3'*  *GC GCC CCGGC CCUCGCU*  *CG CGG GGCCG GGAGUGA*  *miRNA 3' U C GA 5'* |  |
| bHLH112 | *osa-miR531a* | *Position: 1*  *target 5' G GAUCAUCAGAUGAU C 3'*  *AUGG G GCACGCAGCUCCGGCGG*  *UACC C CGUGCGUCGGGGCCGCU*  *miRNA 3' G C 5'* |  |
| bHLH113 | *osa-miR5075* | *Position: 672*  *target 5' A C C G 3'*  *GG ACG CGGCGGCGGAGGA*  *CC UGC GCCGCUGCCUCUU*  *miRNA 3' CG C 5'* |  |

| **Supplementary Table S6**: Predicted microRNA-TF in Arabidopsis- hybrid position, graph and mfe obtained using psRNATarget [37] and RNAhybrid [38]. | | | |
| --- | --- | --- | --- |
| TF name | *miRNA name* | Position | Mfe |
| NF-Y B-3 | *osa-miR5075* | *Position: 31*  *target 5' G C 3'*  *GCGG CGGCGGCGGCGGAGG*  *CGCC GCCGCCGCUGCCUCU*  *miRNA 3' U U 5'* |  |
| NF-Y B-4 | *osa-miR5833* | *Position: 40*  *target 5' A A GAGCGG G 3'*  *GCC CGA GAGCCCGAGGGGGG*  *CGG GCU CUCGGGCUCCUCCU*  *miRNA 3' A 5'* |  |
| NF-Y B-9 | *osa-miR531a,c* | *Position: 48*  *target 5' C GA GCCG C 3'*  *AUGGCGGCG GCAGCU CCGGCGG*  *UACCGCCGU CGUCGG GGCCGCU*  *miRNA 3' G C 5'* |  |

| **Supplementary Table S6**: Predicted microRNA-TF in Arabidopsis- hybrid position, graph and mfe obtained using psRNATarget [37] and RNAhybrid [38]. | | | |
| --- | --- | --- | --- |
| TF name | *miRNA name* | Position | Mfe |
| NF-Y C-2 | *osa-miR5484* | *Position: 461*  *target 5' C G 3'*  *CGGCAGCGCGCUCGG*  *GUUGUCGCGCGAGCC*  *miRNA 3' AUUA AA 5'* |  |
| bZIP17 | *osa-miR5075* | *Position: 708*  *target 5' G C 3'*  *GCGG CGGCGGCGGCGGAGG*  *CGCC GCCGCCGCUGCCUCU*  *miRNA 3' U U 5'* |  |
| TCP21 | *osa-miR2925* | *Position: 569*  *target 5' A AG C 3'*  *GCGGAGC CGCGGCCGCCG*  *UGCUUCG GCGCCGGCGGU*  *miRNA 3' G 5'* |  |
