# Supplementary material for: Cold-responsive transcription factors in Arabidopsis and rice: A regulatory network analysis using array data and gene co-expression network
Source: PLoS One. 2023 Jun 8;18(6):e0286324. doi: 10.1371/journal.pone.0286324 (PMC10249815; doi:10.1371/journal.pone.0286324)
Supplement: S7 Table — (DOCX) [file pone.0286324.s007.docx]

| Supplementary Table S7: Phytohormonal control of TFs obtained from Plant TFDB [32]. | | | |
| --- | --- | --- | --- |
| TF name | Annotation | Phytohormone | Reference |
| ANT | AT4G37750 | - | - |
| ERF 4 | AT3G15210 | abscisic acid, ethylene | Yang et al., 2005 [133] |
| ERF 5 | AT5G47230 | Ethylene, auxin | **Illgen et al., 2020 [134]** |
| ERF13 | At2g44840 | ethylene | [Riechmann and](https://www.uniprot.org/uniprot/?query=author:%22Riechmann+J.L.%22&sort=score)  [Meyerowitz](https://www.uniprot.org/uniprot/?query=author:%22Meyerowitz+E.M.%22&sort=score) , 1998[135] |
| ERF38 | AT2G35700 | ethylene | - |
| ERF73 | AT1G72360 | ethylene | - |
| ERF74-RAP2-12 | AT1G53910 | ethylene | - |
| ERF98 | AT3G23230 | ethylene | - |
| ERF113 | AT5G13330 | abscisic acid, ethylene, jasmonic acid, salicylic acid | **Krishnaswamy et al., 2011[136]** |
| DREB 1A | AT4G25480 | salicylic acid | Miura and Ohta, 2010 [137] |
| DREB 1B/CBF1 | AT4G25490 | salicylic acid | Shi H, et al., 2014 [138] |
| MYB57 | AT3G01530 | [gibberellic acid](https://www.ebi.ac.uk/QuickGO/term/GO:0009740), [jasmonic acid](https://www.ebi.ac.uk/QuickGO/term/GO:0009867) | **Cheng et al., 2009 [139]** |
| MYB59 | AT5G59780 | auxin, ethylene, gibberellin, jasmonic acid, salicylic acid | Hickman et al.2015 **[140]** |
| bHLH16/ UNE10 | AT4G00050 | - | - |
| bHLH35 | AT5G57150 | - | - |
| bHLH 59/ UNE12 | AT4G02590 | - | - |
| bHLH79 | AT5G62610 | - | - |
| bHLH102/BIM2 | AT1G69010 | - | - |
| bHLH105/ ILR3 | AT5G54680 | - | - |
| BHLH116/ICE1 | AT3G26744 | - | - |
| bHLH128 | AT1G05805 | - | - |
| bHLH129 | AT2G43140 | - | - |
| bHLH137 | AT5G50915 | - | - |
| bHLH148 | AT3G06590 | - | - |
| NFYA-4 | AT2G34720 | - | - |
| NFYA-10 | AT5G06510 | - | - |
| bZIP20/TGA2/bzip8 rice | AT5G06950 | salicylic acid | [Kang](https://www.uniprot.org/uniprot/?query=author:%22Kang+H.G.%22&sort=score) and [Klessig, 2005](https://www.uniprot.org/uniprot/?query=author:%22Klessig+D.F.%22&sort=score) **[141]** |
| bZIP45/TGA6 | AT3G12250 | salicylic acid | Zhou et al., 2000 **[142]** |
| bZIP 60 | AT1G42990 | salicylic acid |  |
| GATA 11 | AT1G08010 | - | - |
| GATA 22 | AT4G26150 | cytokinin | Ranftl et al., 2016 **[143]** |
| GATA 23 | AT5G26930 | - | - |
| HSF A-3 | AT5G03720 | - | - |
| HSF A-9 | AT5G54070 | - | - |
| HSF B-2b | AT4G11660 | - | - |
| HSF B4 | AT1G46264 | - | - |
| WRKY1/ZAP1 | AT2G04880 | salicylic acid, auxin-activated signaling pathway | Duan et al., 2007 **[144]** |
| 116PLT2 | At1g51190 | ethylene-activated signaling pathway | - |
| ERF39 | AT4G16750 | ethylene-activated signaling pathway | - |
| ERF54 | AT4G28140 | ethylene-activated signaling pathway | - |
| MYB5 | AT3G13540 | - | - |
| MYB37/RAX1 | AT5G23000 | - | - |
| MYB38/RAX2 | AT2G3689 | abscisic acid, gibberellin, jasmonic acid, salicylic acid | - |
| MYB44 | AT5G67300 | abscisic acid, auxin ethylene | - |
| MYB84/RAX3 | AT3G49690 | salicylic acid | - |
| bHLH112 | AT1G61660 | abscisic acid, | **Liu et al., 2015 [145]** |
| bHLH113 | AT3G19500 | - | - |
| NF-Y B-3 | AT4G14540 | - | - |
| NF-Y B-4 | AT1G09030 | - | - |
| NF-Y B-9 | AT1G21970 | - | - |
| NF-Y C-2 | AT1G56170 | - | - |
| bZIP17 | AT2G40950 | - | - |
| TCP21 | AT5G08330 | - | - |

**References:**

133.[Yang Z](https://www.uniprot.org/uniprot/?query=author:%22Yang+Z.%22&sort=score), [Tian L](https://www.uniprot.org/uniprot/?query=author:%22Tian+L.%22&sort=score), [Latoszek-Green M](https://www.uniprot.org/uniprot/?query=author:%22Latoszek-Green+M.%22&sort=score), [Brown D](https://www.uniprot.org/uniprot/?query=author:%22Brown+D.%22&sort=score), [Wu K.](https://www.uniprot.org/uniprot/?query=author:%22Wu+K.%22&sort=score) **Arabidopsis ERF4 is a transcriptional repressor capable of modulating ethylene and abscisic acid responses. Plant Mol. Biol. 2005;58:585-596.**

134.[Illgen S](https://www.uniprot.org/uniprot/?query=author:%22Illgen+S.%22&sort=score), [Zintl S](https://www.uniprot.org/uniprot/?query=author:%22Zintl+S.%22&sort=score), [Zuther E](https://www.uniprot.org/uniprot/?query=author:%22Zuther+E.%22&sort=score), [Hincha DK](https://www.uniprot.org/uniprot/?query=author:%22Hincha+D.K.%22&sort=score), [Schmulling T.](https://www.uniprot.org/uniprot/?query=author:%22Schmulling+T.%22&sort=score) **Characterisation of the ERF102 to ERF105 genes of Arabidopsis thaliana and their role in the response to cold stress.** [Plant Mol Biol. 2020;103:303-320.](http://dx.doi.org/10.1007/s11103-020-00993-1)

135. Riechmann JL, Meyerowitz EM. The AP2/EREBP family of plant transcription factors. Biol Chem. 1998 Jun;379(6):633-46. doi: 10.1515/bchm.1998.379.6.633. PMID: 9687012.

136.[Krishnaswamy S](https://www.uniprot.org/uniprot/?query=author:%22Krishnaswamy+S.%22&sort=score), [Verma S](https://www.uniprot.org/uniprot/?query=author:%22Verma+S.%22&sort=score), [Rahman MH](https://www.uniprot.org/uniprot/?query=author:%22Rahman+M.H.%22&sort=score), [Kav NN.](https://www.uniprot.org/uniprot/?query=author:%22Kav+N.N.%22&sort=score) **Functional characterization of four APETALA2-family genes (RAP2.6, RAP2.6L, DREB19 and DREB26) in Arabidopsis.** [Plant Mol. Biol. 2011;75:107-127.](http://dx.doi.org/10.1007/s11103-010-9711-7)

137.Miura K, Ohta M. [SIZ1, a small ubiquitin-related modifier ligase, controls cold signaling through regulation of salicylic acid accumulation.](http://www.ncbi.nlm.nih.gov/pubmed/19959255) J. Plant Physiol. 2010;167(7): p. 555-60.

138.Shi H, Wang X, Ye T, Chen F, Deng J, Yang P, Zhang Y, Chan Z. The Cysteine2/Histidine2-Type Transcription Factor ZINC FINGER OF ARABIDOPSIS THALIANA6 Modulates Biotic and Abiotic Stress Responses by Activating Salicylic Acid-Related Genes and C-REPEAT-BINDING FACTOR Genes in Arabidopsis. Plant Physiol. 2014;165(3):1367-1379. doi: 10.1104/pp.114.242404.

139.Cheng H, Song S, Xiao L, Soo HM, Cheng Z, Xie D, Peng J. Gibberellin acts through jasmonate to control the expression of MYB21, MYB24, and MYB57 to promote stamen filament growth in Arabidopsis. PLoS Genet. 2009;5(3):e1000440. doi: 10.1371/journal.pgen.1000440.

140.Hickman R, Van Verk M, Van Dijken A, Pereira Mendes M, Vroegop-Vos I, Caarls L, Steenbergen M, Van der Nagel I, Wesselink G, Jironkin A, Talbot A, Rhodes J, De Vries M, Schuurink R, Denby K, Pieterse C, Van Wees S. Architecture and Dynamics of the Jasmonic Acid Gene Regulatory Network. The Plant Cell*.* 2017;29(9):2086–2105.[doi.org/10.1105/tpc.16.00958](https://doi.org/10.1105/tpc.16.00958).
141.[Kang HG](https://www.uniprot.org/uniprot/?query=author:%22Kang+H.G.%22&sort=score), [Klessig DF.](https://www.uniprot.org/uniprot/?query=author:%22Klessig+D.F.%22&sort=score) **Salicylic acid-inducible Arabidopsis CK2-like activity phosphorylates TGA2.** [Plant Mol Biol. 2005;57:541-557.](http://dx.doi.org/10.1007/s11103-005-0409-1)

142.Zhou JM, Trifa Y, Silva H, Pontier D, Lam E, Shah J, Klessig DF. NPR1 differentially interacts with members of the TGA/OBF family of transcription factors that bind an element of the PR-1 gene required for induction by salicylic acid. Mol Plant Microbe Interact. 2000;13(2):191-202. doi: 10.1094/MPMI.2000.13.2.191.

143.Ranftl QL ,Bastakis E, Klermund C, Schwechheimer C. [LLM-Domain Containing B-GATA Factors Control Different Aspects of Cytokinin-Regulated Development in Arabidopsis thaliana.](http://www.ncbi.nlm.nih.gov/pubmed/26829982) Plant Physiol. 2016; 170(4):2295-311.

144.[Duan MR](https://www.uniprot.org/uniprot/?query=author:%22Duan+M.-R.%22&sort=score), [Nan J](https://www.uniprot.org/uniprot/?query=author:%22Nan+J.%22&sort=score), [Liang YH](https://www.uniprot.org/uniprot/?query=author:%22Liang+Y.-H.%22&sort=score), [Mao P](https://www.uniprot.org/uniprot/?query=author:%22Mao+P.%22&sort=score), [Lu L](https://www.uniprot.org/uniprot/?query=author:%22Lu+L.%22&sort=score), [Li L](https://www.uniprot.org/uniprot/?query=author:%22Li+L.%22&sort=score), [Wei C](https://www.uniprot.org/uniprot/?query=author:%22Wei+C.%22&sort=score), [Lai L](https://www.uniprot.org/uniprot/?query=author:%22Lai+L.%22&sort=score), [Li Y](https://www.uniprot.org/uniprot/?query=author:%22Li+Y.%22&sort=score), [Su XD.](https://www.uniprot.org/uniprot/?query=author:%22Su+X.-D.%22&sort=score) **DNA binding mechanism revealed by high resolution crystal structure of Arabidopsis thaliana WRKY1 protein.** [Nucleic Acids Res. 2007;35:1145-1154.](http://dx.doi.org/10.1093/nar/gkm001)

145.[Liu Y](https://www.uniprot.org/uniprot/?query=author:%22Liu+Y.%22&sort=score), [Ji X](https://www.uniprot.org/uniprot/?query=author:%22Ji+X.%22&sort=score), [Nie X](https://www.uniprot.org/uniprot/?query=author:%22Nie+X.%22&sort=score), [Qu M](https://www.uniprot.org/uniprot/?query=author:%22Qu+M.%22&sort=score), [Zheng L](https://www.uniprot.org/uniprot/?query=author:%22Zheng+L.%22&sort=score), [Tan Z](https://www.uniprot.org/uniprot/?query=author:%22Tan+Z.%22&sort=score), [Zhao H](https://www.uniprot.org/uniprot/?query=author:%22Zhao+H.%22&sort=score), [Huo L](https://www.uniprot.org/uniprot/?query=author:%22Huo+L.%22&sort=score), [Liu S](https://www.uniprot.org/uniprot/?query=author:%22Liu+S.%22&sort=score), [Zhang B](https://www.uniprot.org/uniprot/?query=author:%22Zhang+B.%22&sort=score), [Wang Y.](https://www.uniprot.org/uniprot/?query=author:%22Wang+Y.%22&sort=score) **Arabidopsis AtbHLH112 regulates the expression of genes involved in abiotic stress tolerance by binding to their E-box and GCG-box motifs.** [New Phytol. 2015;207:692-709.](http://dx.doi.org/10.1111/nph.13387)
