# Supplementary material for: Cold-responsive transcription factors in Arabidopsis and rice: A regulatory network analysis using array data and gene co-expression network
Source: PLoS One. 2023 Jun 8;18(6):e0286324. doi: 10.1371/journal.pone.0286324 (PMC10249815; doi:10.1371/journal.pone.0286324)
Supplement: S8 Table — (DOCX) [file pone.0286324.s008.docx]

|  | **Supplementary Table S8**: Gene ontology results of co-expressed genes of up- and down-regulated TFs in rice and Arabidopsis using PANTHER **[45]**. | | | | | |
| --- | --- | --- | --- | --- | --- | --- |
| **Plant** | | **Category** | **Description** | **GO number** | **co-expressed genes of up-regulated TFs*** | **co-expressed genes of down-regulated TFs*** |
| Rice | | Biological process | cellular process | 0009987 | 34.3% | 34.1% |
|  |  |  | biological phase | 0044848 | 0.0% | 1.1% |
|  |  |  | reproductive process | 0022414 | 0.6% | 1.4% |
|  |  |  | localization | 0051179 | 3.9% | 4.0% |
|  |  |  | interspecies interaction between organisms | 0044419 | 0.6% | 0.3% |
|  |  |  | reproduction | 0000003 | 0.6% | 1.4% |
|  |  |  | biological regulation | 0065007 | 16.6% | 14.8% |
|  |  |  | response to stress | 0006950 | 10.2% | 9.4% |
|  |  |  | signaling | 0023052 | 3.5% | 3.7% |
|  |  |  | developmental process | 0032502 | 0.6% | 1.7% |
|  |  |  | rhythmic process | 0048511 | 1.3% | 0.0 |
|  |  |  | multicellular organismal process | 0032501 | 26.3% | 0.6% |
|  |  |  | metabolic process | 0008152 | 17.5% | 27.6% |
|  |  |  | immune system process | 0002376 | 0.1% | 0.0% |
|  |  | Molecular function | translation regulator activity | 0045182 | 0.5% | 0.5% |
|  |  |  | molecular adaptor activity | 0060090 | 0.3% | 0.5% |
|  |  |  | Binding | 0005488 | 37.9% | 34.6% |
|  |  |  | molecular function regulator | 0098772 | 14.9% | 16.8% |
|  |  |  | catalytic activity | 0003824 | 41.3% | 42.7% |
|  |  |  | transporter activity | 0005215 | 4.5% | 3.2% |
|  |  |  | molecular transducer activity | 0060089 | 0.0% | 1.6% |
|  |  | Cellular component | cellular anatomical entity | 0110165 | 52.4% | 51.4% |
|  |  |  | protein-containing complex | 0032991 | 4.8% | 7.9% |
|  |  |  | intracellular | 0005622 | 42.8% | 40.8% |
| Arabidopsis | | Biological process | cellular process | 0009987 | 32.0% | 32.9% |
|  |  |  | reproductive process | 0022414 | 0.9% | 0.8% |
|  |  |  | localization | 0051179 | 5.6% | 3.3% |
|  |  |  | interspecies interaction between organisms | 0044419 | 0.9% | 0.0 |
|  |  |  | reproduction | 0000003 | 0.9% | 0.8% |
|  |  |  | biological regulation | 0065007 | 16.1% | 18.9% |
|  |  |  | response to stress | 0006950 | 11.7% | 9.9% |
|  |  |  | signaling | 0023052 | 3.1% | 3.7% |
|  |  |  | developmental process | 0032502 | 2.4% | 1.6% |
|  |  |  | rhythmic process | 0048511 | 0.3% | 0.4% |
|  |  |  | multicellular organismal process | 0032501 | 2.6% | 0.8% |
|  |  |  | metabolic process | 0008152 | 23.1% | 26.7% |
|  |  |  | immune system process | 0002376 | 0.3% | 0.0 |
|  |  | Molecular function | molecular adaptor activity | 0060090 | 0.3% | 0.0 |
|  |  |  | Binding | 0005488 | 37.8% | 41.8% |
|  |  |  | molecular function regulator | 0098772 | 17.0% | 22.4% |
|  |  |  | catalytic activity | 0003824 | 34.4% | 28.4% |
|  |  |  | transporter activity | 0005215 | 8.8% | 7.5% |
|  |  |  | molecular transducer activity | 0060089 | 1.7% | 0.0 |
|  |  | Cellular component | cellular anatomical entity | 0110165 | 51.8% | 48.8% |
|  |  |  | protein-containing complex | 0032991 | 5.8% | 6.1% |
|  |  |  | intracellular | 0005622 | 42.3% | 45.1% |

*The percent of genes classified to this category over total number of class hits.
