# Supplementary material for: Cold-responsive transcription factors in Arabidopsis and rice: A regulatory network analysis using array data and gene co-expression network
Source: PLoS One. 2023 Jun 8;18(6):e0286324. doi: 10.1371/journal.pone.0286324 (PMC10249815; doi:10.1371/journal.pone.0286324)
Supplement: S9 Table — (DOCX) [file pone.0286324.s009.docx]

| **Supplementary Table S9**: Phytohormone- and abiotic stress- related *Cis*- elements in promoter regions of rice “R” and Arabidopsis “A” TFs. | | | | | | | | | | | | | | | | | | | | | | | | |
| --- | --- | --- | --- | --- | --- | --- | --- | --- | --- | --- | --- | --- | --- | --- | --- | --- | --- | --- | --- | --- | --- | --- | --- | --- |
|  | salicylic acid | | gibberellin- | | abscisic acid | | drought- | | light | | auxin | | low-temperature r | | anaerobic | | MeJA | | circadian | | anoxic | | defense and stress | |
| TF name | R | A | R | A | R | A | R | A | R | A | R | A | R | A | R | A | R | A | R | A | R | A | R | A |
| ANT | * |  | * | * | * |  | * | * | * |  |  | * |  |  |  | * |  |  |  |  |  |  |  |  |
| ERF 4 | * |  |  |  |  | * |  |  | * | * |  |  |  |  |  | * | * | * |  |  |  |  | * | * |
| ERF 5 |  |  |  | * |  |  | * |  | * | * |  | * |  |  | * |  | * |  |  |  |  |  |  | * |
| ERF13 |  |  |  |  | * |  |  |  | * | * | * | * |  |  | * |  | * | * |  | * |  |  |  |  |
| ERF38 |  |  |  | * | * | * |  |  | * | * |  |  |  | * | * | * | * | * |  |  |  |  |  |  |
| ERF73 | * |  |  |  | * |  | * |  |  |  | * | * | * |  | * |  |  | * |  |  |  |  |  |  |
| ERF74 | * |  |  |  |  | * |  |  | * | * | * | * |  |  |  | * | * | * | * | * |  |  | * |  |
| ERF98 |  |  |  |  | * | * | * |  | * | * |  | * |  |  |  |  | * | * |  |  |  |  |  |  |
| ERF113 |  |  |  | * | * | * | * |  | * | * |  |  |  |  | * | * | * | * |  |  |  |  |  | * |
| DREB 1A |  | * |  |  | * | * | * |  | * | * |  | * |  |  | * | * | * | * |  |  |  |  |  |  |
| DREB 1B |  |  |  |  | * | * |  |  | * | * |  |  |  |  | * |  | * |  |  |  |  |  |  |  |
| MYB57 |  | * |  | * |  |  |  |  | * | * |  | * |  |  |  |  | * | * |  |  |  |  |  |  |
| MYB59 |  | * |  |  |  |  |  |  | * | * |  |  |  |  |  |  |  |  |  |  |  |  |  | * |
| bHLH16 |  |  | * |  | * | * |  |  | * | * |  |  |  |  |  |  | * | * |  | * | * |  | * | * |
| bHLH35 |  |  | * | * |  | * |  |  | * | * |  |  |  | * |  | * |  | * |  |  |  |  |  |  |
| bHLH 59 | * | * |  |  |  |  |  | * | * | * | * | * |  |  |  | * | * | * |  |  |  |  |  |  |
| bHLH79 |  | * |  |  | * | * |  | * | * | * |  |  |  |  |  | * | * | * |  | * |  |  |  |  |
| bHLH102 |  |  | * | * |  | * | * |  | * | * |  |  |  | * | * | * |  | * |  |  |  |  |  |  |
| bHLH105 |  | * |  |  |  | * |  |  | * | * |  |  |  | * | * | * |  |  |  |  |  |  |  | * |
| bHLH116 | * | * |  | * |  |  |  |  | * | * |  |  |  | * |  | * | * | * |  |  |  |  |  |  |
| bHLH128 |  |  |  |  | * | * |  |  | * | * |  |  |  | * | * | * | * | * |  | * |  |  |  | * |
| bHLH129 | * |  |  |  | * | * | * | * | * | * |  |  |  |  |  | * | * |  | * |  |  |  |  | * |
| bHLH137 | * |  |  |  |  | * | * | * |  | * |  |  |  | * | * | * |  |  | * |  |  |  |  | * |
| bHLH148 | * |  |  |  | * |  |  |  | * |  |  |  |  |  | * |  | * |  |  |  |  |  |  |  |
| NFYA-4 | * |  |  |  |  | * | * |  | * | * | * |  | * |  |  | * | * | * |  | * | * |  | * |  |
| NFYA-10 |  |  |  |  |  |  | * |  | * | * |  |  | * | * | * |  |  | * |  |  | * |  |  | * |
| bZIP20 |  | * |  | * |  |  |  |  | * | * |  |  |  | * |  | * |  | * |  |  |  |  |  |  |
| bZIP45 |  |  | * |  | * |  |  | * | * | * |  |  |  |  |  | * | * | * |  |  | * |  | * |  |
| bZIP 60 |  |  |  |  | * |  | * |  | * | * |  |  | * |  | * | * | * | * |  |  |  |  |  |  |
| GATA11 | * |  |  |  |  |  | * | * | * | * |  |  |  |  |  |  | * | * |  | * |  |  |  |  |
| GATA22 |  |  |  |  | * | * | * |  | * | * |  |  | * |  | * | * | * |  |  |  | * |  |  | * |
| GATA23 |  | * |  |  | * |  |  | * | * | * |  |  |  |  | * | * | * |  |  |  | * |  |  |  |
| HSF A-3 |  | * | * | * | * | * |  |  | * | * | * |  | * | * |  | * | * | * |  |  | * |  |  |  |
| HSF A-9 |  |  |  |  | * |  |  |  | * | * | * | * |  |  |  | * | * | * |  |  |  |  | * |  |
| HSF B-2b |  |  |  |  | * |  | * |  | * | * |  | * |  |  |  |  | * | * |  |  |  | * |  |  |
| HSF B4 |  |  |  |  |  |  |  |  | * | * |  | * |  |  |  |  |  | * |  |  |  |  |  |  |
| WRKY1 |  |  |  | * | * |  | * |  | * | * |  | * |  |  |  | * | * |  |  |  | * |  |  | * |
| PLT2 |  | * | * |  | * | * |  |  | * | * |  | * | * |  | * | * | * |  |  |  | * |  |  |  |
| ERF39 |  |  | * |  | * | * |  |  | * | * |  |  |  | * |  | * |  |  |  |  | * |  |  |  |
| ERF54 |  | * |  | * | * | * | * |  | * | * |  |  |  |  |  |  |  | * |  | * | * |  |  |  |
| MYB5 |  | * |  | * | * | * |  |  | * | * | * |  |  |  |  | * | * |  |  |  |  |  |  | * |
| MYB37 |  |  |  |  | * |  | * |  | * | * |  |  |  |  | * |  | * |  |  |  |  |  |  | * |
| MYB38 |  |  |  | * | * |  |  |  | * | * | * | * |  |  |  | * | * |  |  | * | * |  |  |  |
| MYB44 |  |  |  |  |  | * |  |  | * | * |  | * | * | * |  | * |  |  |  |  |  |  |  |  |
| MYB84 |  |  |  |  |  | * |  | * | * | * |  |  |  |  |  |  | * | * |  | * |  |  |  | * |
| bHLH112 |  |  |  | * |  |  |  | * | * | * |  | * |  |  |  |  |  |  |  |  |  |  |  |  |
| bHLH113 |  | * |  |  |  | * |  |  | * | * |  |  |  |  |  | * | * | * |  |  |  |  |  | * |
| NF-Y B-3 |  |  |  | * | * | * | * |  | * | * |  | * |  | * |  | * |  | * |  |  |  |  |  |  |
| NF-Y B-4 |  |  |  | * |  |  | * |  | * | * |  |  | * |  |  | * |  | * |  | * |  |  |  |  |
| NF-Y B-9 |  | * |  |  | * | * | * |  | * | * |  |  |  |  | * | * | * | * |  |  |  |  | * | * |
| NF-Y C-2 |  | * |  | * | * | * |  |  | * | * | * |  | * |  |  | * | * |  |  |  |  |  |  |  |
| bZIP17 | * |  |  | * | * | * |  |  | * | * |  |  |  |  | * |  |  | * |  | * |  |  | * |  |
| TCP21 |  |  |  |  | * | * |  |  | * | * | * | * |  |  | * | * | * | * |  |  | * |  |  |  |

| **Continued Supplementary Table S9**: cis- elements and related sequences | | |
| --- | --- | --- |
| *cis*-element name | *Cis*-element | sequence |
| cis-acting element involved in salicylic acid responsiveness | TCA-element | TCAGAAGAGG |
| gibberellin-responsive element | P-box | CCTTTTG |
| cis-acting element involved in the abscisic acid responsiveness | ABRE | ACGTG |
| MYB binding site involved in drought-inducibility | MBS | CAACTG |
| cis-acting regulatory element involved in light responsiveness | G-box | TACGTG |
| auxin-responsive element | TGA-element | AACGAC |
| cis-acting element involved in low-temperature responsiveness | LTR | CCGAAA |
| cis-acting regulatory element essential for the anaerobic induction | ARE | AAACCA |
| cis-acting regulatory element involved in the MeJA-responsiveness | TGACG-motif | TGACG |
| cis-acting regulatory element involved in circadian control | circadian | CAAAGATATC |
| enhancer-like element involved in anoxic specific inducibility | GC-motif | CCCCCG |
| cis-acting element involved in defense and stress responsiveness | TC-rich repeats | GTTTTCTTAC |
