# Supplementary material for: Cold-responsive transcription factors in Arabidopsis and rice: A regulatory network analysis using array data and gene co-expression network
Source: PLoS One. 2023 Jun 8;18(6):e0286324. doi: 10.1371/journal.pone.0286324 (PMC10249815; doi:10.1371/journal.pone.0286324)
Supplement: S10 Table — (DOCX) [file pone.0286324.s010.docx]

| **Supplementary Table S10**: Promoter analysis of co-expressed protein kinases genes of each TF in two model plants were obtained from PlantPAN [48] and AGRIS [49] | | | |
| --- | --- | --- | --- |
| TF name | Kinase gene code | Kinase name | TFs binding to *cis*-elements in kinase promoter |
| ERF4 | At1g73500 | MAP kinase kinase 9 | AT-Hook, MYB; ARR-B, Myb/SANT; ARR-B, C2H2, Trihelix, MYB-related, GATA; tify, G2-like, AP2; RAV, B3, ERF, bHLH, bZIP, C2H2, CSD, TCR; CPP, Dof, EIN3; EIL, GATA; tify, MADS box; MIKC, Homeodomain; HD-ZIP, TALE, MADF, ARR-B, SBP, TCP, BES1, WRKY, NF-YB; NF-YA; NF-YC, |
| ERF5 | At4g11280 | 1-aminocyclopropane-1-carboxylic acid (acc) synthase 6 | AT-Hook, MYB; ARR-B, Myb/SANT; ARR-B, C2H2, Trihelix, MYB-related, GATA; tify, G2-like, AP2; RAV, B3, ERF, bHLH, bZIP, C2H2, CSD, TCR; CPP, Dof, EIN3; EIL, GATA; tify, MADS box; MIKC, Homeodomain; HD-ZIP, TALE, MADF, ARR-B, SBP, TCP, BES1, WRKY, NF-YB; NF-YA; NF-YC, NAC; NAM, |
|  | At3g18690 | MAP kinase substrate 1 | AT-Hook, MYB; ARR-B, Myb/SANT; ARR-B, C2H2, Trihelix, MYB-related, GATA; tify, G2-like, AP2; RAV, B3, ERF, bHLH, bZIP, C2H2, CSD, TCR; CPP, Dof, EIN3; EIL, GATA; tify, MADS box; MIKC, Homeodomain; HD-ZIP, TALE, MADF, ARR-B, SBP, TCP, BES1, WRKY, NF-YB; NF-YA; NF-YC, NAC; NAM |
|  | At1g73500 | MAP kinase kinase 9 | AT-Hook, MYB; ARR-B, Myb/SANT; ARR-B, C2H2, Trihelix, MYB-related, GATA; tify, G2-like, AP2; RAV, B3, ERF, bHLH, bZIP, C2H2, CSD, TCR; CPP, Dof, EIN3; EIL, GATA; tify, MADS box; MIKC, Homeodomain; HD-ZIP, TALE, MADF, ARR-B, SBP, TCP, BES1, WRKY, NF-YB; NF-YA; NF-YC, |
| ERF13 | At4g11280 | 1-aminocyclopropane-1-carboxylic acid (acc) synthase 6 | AT-Hook, MYB; ARR-B, Myb/SANT; ARR-B, C2H2, Trihelix, MYB-related, GATA; tify, G2-like, AP2; RAV, B3, ERF, bHLH, bZIP, C2H2, CSD, TCR; CPP, Dof, EIN3; EIL, GATA; tify, MADS box; MIKC, Homeodomain; HD-ZIP, TALE, MADF, ARR-B, SBP, TCP, BES1, WRKY, NF-YB; NF-YA; NF-YC, NAC; NAM |
|  | At1g73500 | MAP kinase kinase 9 | AT-Hook, MYB; ARR-B, Myb/SANT; ARR-B, C2H2, Trihelix, MYB-related, GATA; tify, G2-like, AP2; RAV, B3, ERF, bHLH, bZIP, C2H2, CSD, TCR; CPP, Dof, EIN3; EIL, GATA; tify, MADS box; MIKC, Homeodomain; HD-ZIP, TALE, MADF, ARR-B, SBP, TCP, BES1, WRKY, NF-YB; NF-YA; NF-YC, |
| ERF74 | At5g25350 | EIN3-binding F box protein 2 | AT-Hook, MYB; ARR-B, Myb/SANT; ARR-B, C2H2, Trihelix, MYB-related, GATA; tify, G2-like, AP2; RAV, B3, ERF, bHLH, bZIP, C2H2, CSD, TCR; CPP, Dof, EIN3; EIL, GATA; tify, MADS box; MIKC, Homeodomain; HD-ZIP, TALE, MADF, ARR-B, SBP, TCP, BES1, WRKY, NF-YB; NF-YA; NF-YC, |
| ERF98 | At3g23240 | ethylene response factor 1 | AT-Hook, MYB; ARR-B, Myb/SANT; ARR-B, C2H2, Trihelix, MYB-related, GATA; tify, G2-like, AP2; RAV, B3, ERF, bHLH, bZIP, C2H2, CSD, TCR; CPP, Dof, EIN3; EIL, GATA; tify, MADS box; MIKC, Homeodomain; HD-ZIP, TALE, MADF, ARR-B, SBP, TCP, BES1, WRKY, NF-YB; NF-YA; NF-YC, |
| MYB59 | At1g78290 | Protein kinase superfamily protein |  |
| bHLH129 | AT2G40330 | PYR1-like 6 | AT-Hook, MYB-related, Dof, AP2; ERF, GATA; tify, YB; G2-like, Myb/SANT, bHLH, bZIP, CG-1; CAMTA, TCR; CPP, Dof, EIN3; EIL, Homeodomain; HD-ZIP, WOX, MADF; Trihelix, Myb/SANT; ARR-B, TCP, TBP, BES1, B3; RAV, ZF-HD, NF-YB; NF-YA; NF-YC, Trihelix, ARID; Sox, |
| bZIP60 | At2g38470 | WRKY DNA-binding protein 33 | BHLH, Homeobox, bZIP, WRKY, ARF, ABI3VP1, MYB |
|  | At3g25250 | AGC (cAMP-dependent, cGMP-dependent and protein kinase C) kinase family protein | BHLH, Homeobox, bZIP,WRKY, ARF, HB, MYB-related, MYB, ABI3VP1, LFY |
| ERF39 | At5g07390 | respiratory burst oxidase homolog A | AT-Hook, MYB; ARR-B, Myb/SANT; ARR-B, C2H2, Trihelix, MYB-related, GATA; tify, G2-like, AP2; RAV, B3, ERF, bHLH, bZIP, C2H2, CSD, TCR; CPP, Dof, EIN3; EIL, GATA; tify, MADS box; MIKC, Homeodomain; HD-ZIP, TALE, MADF, ARR-B, SBP, TCP, BES1, WRKY, NF-YB; NF-YA; NF-YC |

| **Supplementary Table S10**: Promoter analysis of co-expressed protein kinases genes of each TF in two model plants were obtained from PlantPAN [48] and AGRIS [49] | | | |
| --- | --- | --- | --- |
| TF name | Kinase gene RAP-ID | Kinase name | TFs binding to cis-elements in kinase promoter |
| ANT | Os06g0203800 | LRR receptor-like serine/threonine-protein kinase ERECTA | AP2; ERF, bHLH, bZIP, B3, C2H2, TCR, E2F, EIN3, GATA, HD-ZIP, Homeodomain; TALE, ZF-HD, Myb/SANT, TCP, SBP, MADS box, MIKC, M-type, TBP, NF-YB, Alpha-amylase, AT-Hook, PsaH |
| ERF73 | Os02g0165100 | Protein kinase-like domain containing protein. | AP2; B3; RAV, bHLH, bZIP, B3; ARF, TCR, EIN3, GATA, Homeodomain; HD-ZIP, WOX, TALE, Myb/SANT, NAC; NAM, SBP, TCP, WRKY, MADS box; MIKC; M-typ, AT-Hook, NF-YB, Alpha-amylase, ERF, LEA_5 |
|  | Os01g0955100 | calcium-binding protein CML | AP2; ERF, AT-Hook, B3; ARF, bHLH, bZIP, B3, C2H2, CG-1, TCR, E2F, GATA, Dof, Homeodomain; HD-ZIP, TALE, MADF, Myb/SANT, NAC; NAM, SBP, TCP, TBP, RAV, PsaH, NF-YB, Alpha-amylase, ERF, BES1, LEA_5 |
|  | Os05g0545300 | Protein kinase-like domain containing protein.mitogen-activated protein kinase kinase kinase 17/18 | AP2, ERF, AT-Hook, bHLH, bZIP, C2H2, E2F, EIN3, GATA, Homeodomain; TALE, HD-ZIP, WOX, ZF-HD, Myb/SANT, NAC; NAM, G2-like, TCP, MADS box; MIKC; M-type, B3, WRKY, TBP, NF-YB, Alpha-amylase, BES1 |
|  | Os10g0392400 | ZIM domain containing protein.jasmonate ZIM domain-containing protein | NA |
| ERF113 | Os03g0285800 | MAP Kinase. | AP2; ERF, AT-Hook, bHLH, bZIP, B3, C2H2, CG-1, TCR, GATA, Homeodomain; HD-ZIP, WOX, ZF-HD, MADF, Myb/SANT, NAC; NAM, SBP, WRKY, TBP, TALE, NF-YB, TCP, Alpha-amylase, BES1 |
|  | Os01g0846300 | Similar to Protein phosphatase 2C. | AP2; ERF, AT-Hook, B3; ARF, bHLH, B3; bZIP, C2H2, CG-1, E2F, GATA, Homeodomain; HD-ZIP, WOX, TALE, MADF, Myb/SANT, NAC; NAM, TCP, RAV, MYB, TBP, TALE, NF-YB, TCP, Alpha-amylase, BES1 |
|  | Os03g0268600 | Similar to Protein phosphatase type 2C. | NA |
|  | Os09g0325700 | Similar to Protein phpsphatase 2C (PP2C) (EC 3.1.3.16). | AP2; ERF, AT-Hook, bHLH, B3; ARF, bZIP, CG-1, TCR, Dof; GATA, E2F, Homeodomain; TALE, ZF-HD, LOB, MADF, Myb/SANT, G2-like, NAC; NAM, SBP, TBP, TCP, RAV, EIN3, NF-YB, Alpha-amylase |
| bHLH116 | Os07g0685700 | Ethylene insensitive 3 family protein. | AP2; ERF, AT-Hook, B3; ARF, bHLH, bZIP, C2H2, GATA, Homeodomain; HD-ZIP, LOB, MADF, Myb/SANT, NAC; NAM, SBP, WRKY, RAV, TBP, PsaH, TALE, LEA_5, NF-YB, TCP, Alpha-amylase, BES1 |
| Bhlh137 | Os06g0203800 | Similar to ERECTA-like kinase 1. | AP2; ERF, bHLH, bZIP, B3, C2H2, TCR, E2F, EIN3, GATA, HD-ZIP, Homeodomain; TALE, ZF-HD, Myb/SANT, TCP, SBP, MADS box, MIKC, M-type, TBP, NF-YB, Alpha-amylase |
| bhlh148 | Os03g0285800 | MAP Kinase. | AP2; ERF, AT-Hook, bHLH, bZIP, B3, C2H2, CG-1, TCR, GATA, Homeodomain; HD-ZIP, WOX, ZF-HD, MADF, Myb/SANT, NAC; NAM, SBP, WRKY, TBP, TALE, NF-YB, TCP, Alpha-amylase, BES1 |
|  | Os05g0545300 | Protein kinase-like domain containing protein. | AP2, ERF, AT-Hook, bHLH, bZIP, C2H2, E2F, EIN3, GATA, Homeodomain; TALE, HD-ZIP, WOX, ZF-HD, Myb/SANT, NAC; NAM, G2-like, TCP, MADS box; MIKC; M-type, B3, WRKY, TBP, NF-YB, Alpha-amylase, BES1 |
|  | Os07g0685700 | Ethylene insensitive 3 family protein. | AP2; ERF, AT-Hook, B3; ARF, bHLH, bZIP, C2H2, GATA, Homeodomain; HD-ZIP, LOB, MADF, Myb/SANT, NAC; NAM, SBP, WRKY, RAV, TBP, PsaH, TALE, LEA_5, NF-YB, TCP, Alpha-amylase, BES1 |
| NFY-A4 | Os01g0846300 | Similar to Protein phosphatase 2C. | AP2; ERF, AT-Hook, B3; ARF, bHLH, B3; bZIP, C2H2, CG-1, E2F, GATA, Homeodomain; HD-ZIP, WOX, TALE, MADF, Myb/SANT, NAC; NAM, TCP, RAV, MYB, TBP, TALE, NF-YB, TCP, Alpha-amylase, BES1 |
|  | Os05g0537400 | Similar to Protein phosphatase 2C. | AP2; ERF, B3; RAV, AT-Hook, bHLH, bZIP, C2H2, CG-1, E2F, EIN3, GATA, Homeodomain; HD-ZIP, ZF-HD, MADF, Myb/SANT, NAC; NAM, TBP, TCP, EIN3, TALE, NF-YB, Alpha-amylase, SBP |
|  | Os09g0325700 | Similar to Protein phosphatase 2C (PP2C) (EC 3.1.3.16). | AP2; ERF, AT-Hook, bHLH, B3; ARF, bZIP, CG-1, TCR, Dof; GATA, E2F, Homeodomain; TALE, ZF-HD, LOB, MADF, Myb/SANT, G2-like, NAC; NAM, SBP, TBP, TCP, RAV, EIN3, NF-YB, Alpha-amylase |
| bZIP45 | Os09g0325700 | Similar to Protein phosphatase 2C (PP2C) (EC 3.1.3.16). | AP2; ERF, AT-Hook, bHLH, B3; ARF, bZIP, CG-1, TCR, Dof; GATA, E2F, Homeodomain; TALE, ZF-HD, LOB, MADF, Myb/SANT, G2-like, NAC; NAM, SBP, TBP, TCP, RAV, EIN3, NF-YB, Alpha-amylase |
|  | Os01g0846300 | Similar to Protein phosphatase 2C. | AP2; ERF, AT-Hook, B3; ARF, bHLH, B3; bZIP, C2H2, CG-1, E2F, GATA, Homeodomain; HD-ZIP, WOX, TALE, MADF, Myb/SANT, NAC; NAM, TCP, RAV, MYB, TBP, TALE, NF-YB, TCP, Alpha-amylase, BES1 |
| GATA33 | Os07g0492000 | Nucleoside diphosphate kinase I (EC 2.7.4.6) (NDK I) (NDP kinase I) (NDPK I). | AP2; ERF, AT-Hook, bHLH, bZIP, GATA, CG-1, Homeodomain; HD-ZIP, WOX, TALE, HD-ZIP, LOB, MADF, Myb/SANT, G2-like, NAC; NAM, SBP, TCP, WRKY, TBP, MADS box; MIKC; M-type, B3; RAV, SBP, NF-YB, Alpha-amylase, BES1 |
| HSFa3 | Os01g0656200 | Protein phosphatase 2C family protein. | AP2; ERF, AT-Hook, TCR, EIN3, AP2; ERF, AT-Hook, bHLH, bZIP, GATA, CG-1, Homeodomain; HD-ZIP, WOX, TALE, HD-ZIP, LOB, MADF, Myb/SANT, G2-like, NAC; NAM, SBP, TCP, WRKY, TBP, MADS box; MIKC; M-type, B3; RAV, SBP, NF-YB, Alpha-amylase, BES1 |

| **Supplementary Table S10**: Promoter analysis of co-expressed protein kinases genes of each TF in two model plants were obtained from PlantPAN [48] and AGRIS [49] | | | |
| --- | --- | --- | --- |
| TF name | Kinase gene RAP ID | Kinase name | TFs binding to cis-elements in kinase promoter |
| WRKY1 | Os05g0343400 | WRKY 53 (Transcription factor WRKY12). | AP2; B3; RAV, AT-Hook, bHLH, bZIP, C2H2, CG-1, TCR, GATA, Dof, Homeodomain; HD-ZIP, WOX, MADF, Myb/SANT, NAC; NAM, WRKY, TBP, SBP, TALE, NF-YB, TCP, Alpha-amylase, ERF, LEA_5E2F, EIN3, |
|  | Os01g0826400 | WRKY 24 (WRKY24). | AP2; B3; RAV, AT-Hook, bHLH, bZIP, C2H2, CG-1, TCR, GATA, Dof, Homeodomain; HD-ZIP, WOX, MADF, Myb/SANT, NAC; NAM, WRKY, TBP, SBP, TALE, NF-YB, TCP, Alpha-amylase, ERF, LEA_5E2F, EIN3, G2-like, NFYB |
| PLT | Os07g0259100 | Similar to Ethylene receptor. | AP2; ERF, AT-Hook, B3; ARF, bHLH, bZIP, B3, C2H2, CG-1, TCR, E2F, GATA, Dof, Homeodomain; HD-ZIP, TALE, MADF, Myb/SANT, NAC; NAM, SBP, TCP, TBP, RAV, PsaH, NF-YB, Alpha-amylase, ERF, BES1, LEA_5 |
| MYB37 | Os04g0691100 | Serine/threonine-protein kinase SAPK5 (EC 2.7.1.37) (Osmotic stress/abscisic acid-activated protein kinase 5). | AP2; B3; RAV, AT-Hook, bHLH, bZIP, C2H2, CG-1, TCR, GATA, Dof, Homeodomain; HD-ZIP, WOX, MADF, Myb/SANT, NAC; NAM, WRKY, TBP, SBP, TALE, NF-YB, TCP, Alpha-amylase, ERF, LEA_5 |
|  |  |  |  |
