# Supplementary material for: Cold-responsive transcription factors in Arabidopsis and rice: A regulatory network analysis using array data and gene co-expression network
Source: PLoS One. 2023 Jun 8;18(6):e0286324. doi: 10.1371/journal.pone.0286324 (PMC10249815; doi:10.1371/journal.pone.0286324)
Supplement: S11 Table — Data was obtained from KEGG [50]. (DOCX) [file pone.0286324.s011.docx]

| **Supplementary Table S11**: Co-expressed genes of TF families in rice and Arabidopsis were active in signal transduction under the control of different hormones. Data was obtained from KEGG [50]. | | | | |
| --- | --- | --- | --- | --- |
| TF name | Number of genes in rice | Number of genes in Arabidopsis | Phytohormone in rice | Phytohormone in Arabidopsis |
| ANT | 5 | 1 | Auxin, cyclin D3 | cyclin D3 |
| ERF | 12 | 23 | ABA, jasmonate | Abscisic acid, Ethylene, jasmonate |
| MYB | 1 | 3 | jasmonate | auxin |
| bHLH | 19 | 6 | Auxin, Ethylene, jasmonate | IAA, jasmonate, Auxin |
| NFY-A | 7 | 1 | Abscisic acid | salicylic acid |
| bZIP | 7 | 6 | salicylic acid, ABA | Auxin, cyclin D3 |
| GATA | 1 | 0 | - | - |
| HSF | 1 | 0 | - | - |
| WRKY | 2 | 0 | - | - |
| PLT | 2 | 4 | ethylene | - |
| ERF-down | 0 | 1 | - | - |
| MYB-down | 3 | 3 | ABA | - |
| bHLH-down | 10 | 3 | Jasmonate | Jasmonate |
| NFYB-down | 1 | 0 | Auxin, ABA | - |
| bzip-down | 1 | 0 | salicylic acid, ABA |  |
| TCP | 0 | 0 | - | - |
