# Supplementary material for: Cold-responsive transcription factors in Arabidopsis and rice: A regulatory network analysis using array data and gene co-expression network
Source: PLoS One. 2023 Jun 8;18(6):e0286324. doi: 10.1371/journal.pone.0286324 (PMC10249815; doi:10.1371/journal.pone.0286324)
Supplement: S12 Table — (DOCX) [file pone.0286324.s012.docx]

| **Supplementary Table S12**: Co-expressed genes of putative cold-responsive TFs in rice and Arabidopsis involved in lipid metabolism. | | | |
| --- | --- | --- | --- |
| TF name | Lipid metabolism | Arabidopsis | Rice |
| ANT | Glycerophospholipid metabolism | 1 | 0 |
|  | Glycerolipid metabolism | 1 | 0 |
|  | Fatty acid elongation | 0 | 1 |
|  |  | 0 | 0 |
| ERF | alpha-Linolenic acid metabolism | 0 | 2 |
|  | Glycerophospholipid metabolism | 1 | 1 |
|  | Fatty acid degradation | 4 | 0 |
|  | Biosynthesis of unsaturated fatty acids | 2 | 0 |
|  | Ether lipid metabolism | 1 | 0 |
|  | Sphingolipid metabolism | 1 | 0 |
|  | Glycerolipid metabolism | 1 | 0 |
|  | Fatty acid biosynthesis | 1 | 0 |
|  |  |  |  |
|  |  |  |  |
| MYB | Glycerolipid metabolism | 0 | 1 |
|  | Fatty acid degradation | 1 |  |
|  | Glycerolipid metabolism | 0 | 1 |
|  |  |  |  |
| bHLH | alpha-Linolenic acid metabolism | 0 | 1 |
|  | Fatty acid degradation | 1 |  |
|  | Biosynthesis of unsaturated fatty acids | 0 | 2 |
|  | Synthesis and degradation of ketone bodies | 1 |  |
|  | Glycerolipid metabolism | 0 | 1 |
|  | Fatty acid elongation | 0 | 3 |
|  | Linoleic acid metabolism | 0 | 1 |
|  | Steroid biosynthesis | 1 |  |
|  |  |  |  |
| GATA33 | Glycerolipid metabolism | 0 | 1 |
|  | Fatty acid degradation | 0 | 1 |
|  | Ether lipid metabolism | 0 | 1 |
|  |  |  |  |
| HSF | alpha-Linolenic acid metabolism | 0 | 1 |
|  | Glycerophospholipid metabolism | 0 | 2 |
|  | Glycerolipid metabolism | 0 | 1 |
|  | Fatty acid biosynthesis | 0 | 1 |
|  |  |  |  |
| WRKY | alpha-Linolenic acid metabolism | 0 | 1 |
|  | Fatty acid biosynthesis | 0 | 1 |
|  | Fatty acid elongation | 0 | 1 |
|  |  |  |  |
| ERF | Cutin, suberine and wax biosynthesis | 0 | 1 |
|  |  |  |  |
| MYB | alpha-Linolenic acid metabolism | 0 | 2 |
|  | Glycerophospholipid metabolism | 0 | 2 |
|  | Fatty acid degradation | 0 | 1 |
|  | Biosynthesis of unsaturated fatty acids | 0 | 1 |
|  | Ether lipid metabolism | 0 | 1 |
|  | Fatty acid elongation | 0 | 1 |
|  | Steroid biosynthesis | 1 | 1 |
|  |  |  |  |
| bHLH | alpha-Linolenic acid metabolism | 0 | 3 |
|  | Glycerophospholipid metabolism | 0 | 1 |
|  | Ether lipid metabolism | 0 | 1 |
|  | Linoleic acid metabolism | 0 | 2 |
|  |  |  |  |
| NFYB | Synthesis and degradation of ketone bodies | 0 | 1 |
|  |  |  |  |
| TCP | alpha-Linolenic acid metabolism | 0 | 1 |
|  | Fatty acid degradation | 0 | 1 |
