# Supplementary material for: Cold-responsive transcription factors in Arabidopsis and rice: A regulatory network analysis using array data and gene co-expression network
Source: PLoS One. 2023 Jun 8;18(6):e0286324. doi: 10.1371/journal.pone.0286324 (PMC10249815; doi:10.1371/journal.pone.0286324)
Supplement: S13 Table — (DOCX) [file pone.0286324.s013.docx]

| **Supplementary Table S13**: Metabolic pathways that co-expressed genes of Arabidopsis TFs are involved in each group. | | |
| --- | --- | --- |
| Group | TFs | Metabolic pathways number of genes |
| **Group 1**  ANT, HSFB2b, HSFA9,  HSFB4 | ANT | \| Metabolic pathways \| 1 \| \| --- \| --- \| \| Biosynthesis of secondary metabolites \| 1 \| \| Carbohydrate metabolism \| 2 \| \| Lipid metabolism \| 2 \| \| Signal transduction \| 1 \| |
|  | HSF | \| Biosynthesis of secondary metabolites \| 1 \| \| --- \| --- \| \| Biosynthesis of secondary metabolites \| 1 \| \| Biosynthesis of cofactors \| 2 \| \| Carbohydrate metabolism \| 2 \| \| Metabolism of cofactors and vitamins \| 3 \| \| Transcription \| 3 \| \| Folding, sorting and degradation \| 10 \| \| Signal transduction \| 3 \| \| Plant-pathogen interaction \| 1 \| \| Oxidative phosphorylation \| 1 \| |
| **Group** **2**  UNE10, UNE12, BIM2 | bHLH | \| Metabolic pathways \| 15 \| \| --- \| --- \| \| Biosynthesis of secondary metabolites \| 14 \| \| Fatty acid metabolism \| 1 \| \| Carbon metabolism \| 1 \| \| Ascorbate and aldarate metabolism \| 1 \| \| 2-Oxocarboxylic acid metabolism \|  \| \| Biosynthesis of amino acids \|  \| \| Biosynthesis of cofactors \| 3 \| \| Carbohydrate metabolism \| 8 \| \| Energy metabolism \| 2 \| \| Lipid metabolism \| 3 \| \| Nucleotide metabolism \| 1 \| \| Amino acid metabolism \| 5 \| \| Metabolism of cofactors and vitamins \| 1 \| \| Metabolism of terpenoids and polyketides \| 4 \| \| Translation \| 2 \| \| Folding, sorting and degradation \| 4 \| \| Membrane transport \| 1 \| \| Signal transduction \| 6 \| \| Transport and catabolism \| 2 \| \| Plant-pathogen interaction \| 3 \| \| Circadian rhythm - plant \| 1 \| \| Oxidative phosphorylation \| 2 \| |

| **Supplementary Table S13**: Metabolic pathways that co-expressed genes of Arabidopsis TFs are involved in each group. | | |
| --- | --- | --- |
| Group | TFs | Metabolic pathways number of genes |
| **Group 3**  NFYA4, NFYC2, NFYB4, NFYA10, bZIP17, bZIP60, NFYB3 | NFYA | \| Metabolic pathways \| 2 \| \| --- \| --- \| \| Biosynthesis of secondary metabolites \| 0 \| \| Biosynthesis of amino acids \| 1 \| \| Carbohydrate metabolism \| 1 \| \| Energy metabolism \| 1 \| \| Amino acid metabolism \| 2 \| \| Metabolism of cofactors and vitamins \| 1 \| \| Metabolism of terpenoids and polyketides \| 0 \| \| Biosynthesis of other secondary metabolites \| 2 \| \| Folding, sorting and degradation \| 0 \| \| Signal transduction \| 1 \| \| Transport and catabolism \| 1 \| \| Circadian rhythm - plant \| 1 \| |
|  | NFYB | \| Metabolic pathways \| 4 \| \| --- \| --- \| \| Biosynthesis of secondary metabolites \| 3 \| \| Biosynthesis of amino acids \| 0 \| \| Biosynthesis of cofactors \| 1 \| \| Carbohydrate metabolism \| 3 \| \| Energy metabolism \| 0 \| \| Lipid metabolism \| 0 \| \| Amino acid metabolism \| 0 \| \| Metabolism of cofactors and vitamins \| 1 \| \| Metabolism of terpenoids and polyketides \| 2 \| \| Biosynthesis of other secondary metabolites \| 0 \| \| Translation \| 2 \| \| Folding, sorting and degradation \| 2 \| \| Plant-pathogen interaction \| 2 \| |
|  | bZIP | \| Metabolic pathways \| 1 \| \| --- \| --- \| \| Amino acid metabolism \| 1 \| \| Transcription \| 2 \| \| Translation \| 2 \| \| Folding, sorting and degradation \| 3 \| \| Signal transduction \| 6 \| \| Transport and catabolism \| 2 \| \| Plant-pathogen interaction \| 3 \| \| Circadian rhythm - plant \| 1 \| |

| **Supplementary Table S13**: Metabolic pathways that co-expressed genes of Arabidopsis TFs are involved in each group. | | |
| --- | --- | --- |
| Group | TFs | Metabolic pathways number of genes |
| **Group 4**  ERF4, ERF5, ERF13, ICE1 | ERF | \| Biosynthesis of secondary metabolites \| 31 \| \| --- \| --- \| \| Amino acid metabolism \| 24 \| \| Signal transduction \| 23 \| \| Folding, sorting and degradation \| 13 \| \| Carbohydrate metabolism \| 11 \| \| Lipid metabolism \| 11 \| |
|  | bHLH116/ICE1 | \| Carbohydrate metabolism \| 4 \| \| --- \| --- \| \| Biosynthesis of secondary metabolites \| 3 \| \| Signal transduction \| 3 \| |
