# Supplementary material for: Cold-responsive transcription factors in Arabidopsis and rice: A regulatory network analysis using array data and gene co-expression network
Source: PLoS One. 2023 Jun 8;18(6):e0286324. doi: 10.1371/journal.pone.0286324 (PMC10249815; doi:10.1371/journal.pone.0286324)
Supplement: S14 Table — (DOCX) [file pone.0286324.s014.docx]

| **Supplementary Table 14**: Metabolic pathways which co-expressed genes of rice TFs are involved in each group. | | |
| --- | --- | --- |
| Group | TFs | Metabolic pathways Number of genes |
| **Group 1**  DREB1B, ERF24 | ERF | \| Carbohydrate metabolism \| 2 \| \| --- \| --- \| \| Lipid metabolism \| 3 \| \| Amino acid metabolism \| 2 \| \| Metabolism of terpenoids and polyketides \| 6 \| \| Biosynthesis of secondary metabolites \| 6 \| \| Folding, sorting and degradation \| 6 \| \| Signal transduction \| 12 \| \| Plant-pathogen interaction \| 8 \| \| Circadian rhythm - plant \| 3 \| |
| **Group 2**  HSF8, HSF21, HSF19, HSFA3 | HSF | \| Biosynthesis of secondary metabolites \| 3 \| \| --- \| --- \| \| Fatty acid metabolism \| 1 \| \| Biosynthesis of secondary metabolites \| 3 \| \| Fatty acid metabolism \| 1 \| \| Biosynthesis of cofactors \| 2 \| \| Carbohydrate metabolism \| 1 \| \| Energy metabolism \| 1 \| \| Lipid metabolism \| 5 \| \| Nucleotide metabolism \| 1 \| \| Metabolism of other amino acids \| 3 \| \| Metabolism of cofactors and vitamins \| 2 \| \| Glycan biosynthesis and metabolism \| 1 \| \| Transcription \| 5 \| \| Translation \| 1 \| \| Folding, sorting and degradation \| 22 \| \| Signal transduction \| 8 \| \| Plant-pathogen interaction \| 4 \| \| Circadian rhythm - plant \| 3 \| \| Chemical carcinogenesis- reactive oxygen species \| 1 \| |
| **Group 3**  NFYB3, HAP5C | NFYB | \| Metabolic pathways \| 20 \| \| --- \| --- \| \| Biosynthesis of secondary metabolites \| 20 \| \| Biosynthesis of amino acids \| 1 \| \| Biosynthesis of cofactors \| 1 \| \| Carbohydrate metabolism \| 2 \| \| Energy metabolism \| 31 \| \| Lipid metabolism \| 1 \| \| Amino acid metabolism \| 3 \| \| Metabolism of cofactors and vitamins \| 2 \| \| Metabolism of terpenoids and polyketides \| 2 \| \| Biosynthesis of other secondary metabolites \| 2 \| \| Translation \| 1 \| \| Replication and repair \| 1 \| \| Signal transduction \| 1 \| \| Transport and catabolism \| 1 \| \| Chemical carcinogenesis- reactive oxygen species \| 1 \| |

| **Supplementary Table 14**: Metabolic pathways which co-expressed genes of rice TFs are involved in each group. | | |
| --- | --- | --- |
| Group | TFs | Metabolic pathways Number of genes |
| **Group** **4**  bHLH148, bHLH6 | bHLH | \| Biosynthesis of secondary metabolites \| 27 \| \| --- \| --- \| \| Carbohydrate metabolism \| 22 \| \| Amino acid metabolism \| 11 \| \| Signal transduction \| 19 \| |
