# Supplementary material for: Cold-responsive transcription factors in Arabidopsis and rice: A regulatory network analysis using array data and gene co-expression network
Source: PLoS One. 2023 Jun 8;18(6):e0286324. doi: 10.1371/journal.pone.0286324 (PMC10249815; doi:10.1371/journal.pone.0286324)
Supplement: S1 Fig — (DOCX) [file pone.0286324.s015.docx]

**Supplementary Figure S1**: Metabolic pathway of co-expressed genes of down-regulated TFs in rice and Arabidopsis.
