# Supplementary figures and images for: Cold-responsive transcription factors in Arabidopsis and rice: A regulatory network analysis using array data and gene co-expression network
Source: PLoS One. 2023 Jun 8;18(6):e0286324. doi: 10.1371/journal.pone.0286324 (PMC10249815; doi:10.1371/journal.pone.0286324)

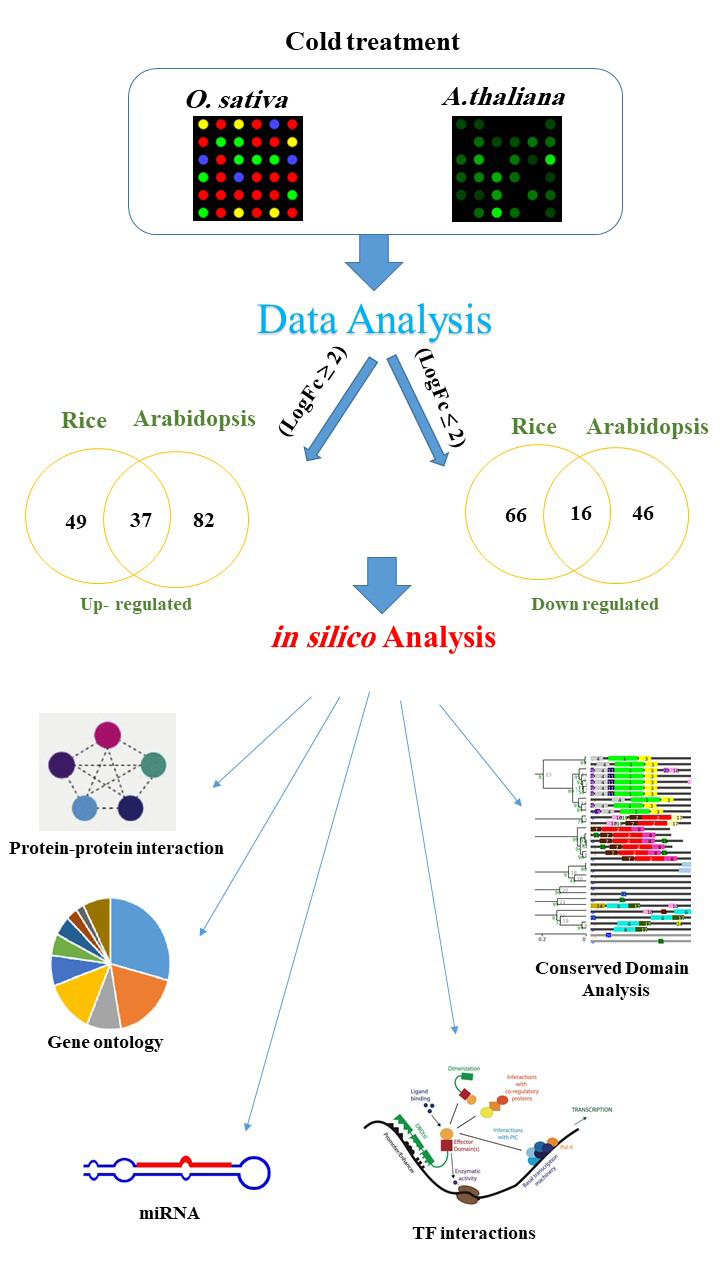

Supplement: S1 Graphical abstract — Common TFs were separated. In silico analysis was applied including conserved domain analysis, TF interactions, post-transcriptional analysis (miRNAs), co-expression analysis, gene ontology, and PPI Network. (TIF) [file pone.0286324.s016.tif]
